# *ABCG2* Gene and ABCG2 Protein Expression in Colorectal Cancer—In Silico and Wet Analysis

**DOI:** 10.3390/ijms241310539

**Published:** 2023-06-23

**Authors:** Aleksandra Sałagacka-Kubiak, Dawid Zawada, Lias Saed, Radzisław Kordek, Agnieszka Jeleń, Ewa Balcerczak

**Affiliations:** 1 Department of Pharmaceutical Biochemistry and Molecular Diagnostics, Medical University of Lodz, 92-213 Lodz, Poland; dawid.zawada2@gmail.com (D.Z.); lias.saed@stud.umed.lodz.pl (L.S.); agnieszka.jelen@umed.lodz.pl (A.J.);; 2 Department of Pathology, Medical University of Lodz, 92-213 Lodz, Poland; radzislaw.kordek@umed.lodz.pl

**Keywords:** ABCG2, prognosis, survival, protein–protein interaction network, immunohistochemistry, qPCR

## Abstract

ABCG2 (ATP-binding cassette superfamily G member 2) is a cell membrane pump encoded by the *ABCG2* gene. ABCG2 can protect cells against compounds initiating and/or intensifying neoplasia and is considered a marker of stem cells responsible for cancer growth, drug resistance and recurrence. Expression of the *ABCG2* gene or its protein has been shown to be a negative prognostic factor in various malignancies. However, its prognostic significance in colorectal cancer remains unclear. Using publicly available data, *ABCG2* was shown to be underexpressed in colon and rectum adenocarcinomas, with lower expression compared to both the adjacent nonmalignant lung tissues and non-tumour lung tissues of healthy individuals. This downregulation could result from the methylation level of some sites of the *ABCG2* gene. This was connected with microsatellite instability, weight and age among patients with colon adenocarcinoma, and with tumour localization, population type and age of patients for rectum adenocarcinoma. No association was found between *ABCG2* expression level and survival of colorectal cancer patients. In wet analysis of colorectal cancer samples, neither *ABCG2* gene expression, analysed by RT-PCR, nor ABCG2 protein level, assessed by immunohistochemistry, was associated with any clinicopathological factors or overall survival. An ABCG2-centered protein–protein interaction network build by STRING showed proteins were found to be involved in leukotriene, organic anion and xenobiotic transport, endodermal cell fate specification, and histone methylation and ubiquitination. Hence, ABCG2 underexpression could be an indicator of the activity of certain signalling pathways or protein interactors essential for colorectal carcinogenesis.

## 1. Introduction

Despite the existence of effective screening techniques and the unquestionable advancement in treatment options, colorectal cancer remains the third most common cancer type worldwide. According to GLOBOCAN, the condition was responsible for almost one million deaths in 2020, with nearly two million new cases that year [1]. It also predicted that the number of new cases and the number of deaths will increase by about 70% of today’s values by 2040 [2]. Currently, the major obstacles to the successful management of colorectal cancer are difficulties in accurate prediction of the further course of colorectal cancer and choosing the optimal treatment schedule, as well as predicting the response to applied therapy. Thus, the research efforts focused on new biological markers related to the neoplastic process that can be transferable to clinical practice. 

**Figure 1:**
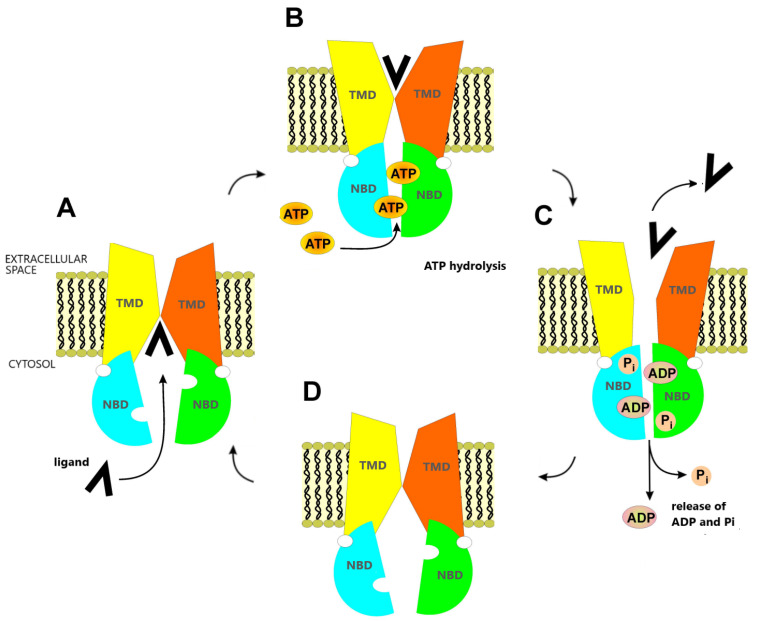
Scheme of the structure and the process of transport across the membrane with the participation of ABCG2. The transport takes place in four closely related stages. (**A**) Transmembrane domains (TMDs) adopt an inward-facing conformation with high affinity to the exported compound, which attaches itself to a special pocket formed by two TMD domains. This induces conformational changes in nucleotide-binding domains (NBDs) so that they increase their affinity for ATP. (**B**) Two ATP molecules are attached, resulting in NBFs approaching each other. This causes the TMDs to adopt an outward-facing conformation. (**C**) Transfer and release of the exported ligand across the membrane. ATP hydrolysis, phosphate and ADP release occur in parallel. (**D**) Relaxation of NBDs, return of TMDs to inward-facing conformation. The transporter is ready to accept the next ligand molecule, and the whole cycle can be repeated [5,6,7,8].

ABCG2 (ATP-binding cassette superfamily G member 2), encoded by the *ABCG2* gene, is an ATP-dependent transporter belonging to the ATP-binding cassette (ABC) protein superfamily. The ABCG2 localized in the cell membrane pumps the drug molecules from the cytoplasm out of the cells (Figure 1). This diminishes access of the drug to the cellular target, and thus reduces the effectiveness of the applied therapy. As ABCG2 is characterized by low substrate specificity, and its substrates include drugs from various therapeutic groups and their metabolites, it may provide resistance to a wide variety of anticancer drugs—a phenomenon termed multidrug resistance (MDR) [3]. Some studies have suggested that the *ABCG2* gene and its protein may act as indicators for the prediction of irinotecan-based therapy outcomes in colorectal cancer patients (reviewed in [4]). 

However, the role of ABCG2 in creating the MDR phenomenon is a manifestation of its physiological properties. ABCG2 is present in placental syncytiotrophoblast cells, epithelium of the intestine, liver tubules, ducts and lobes of the breast, renal proximal tubule cells, adrenal glands, stem cells and endothelium of capillaries and veins. It controls the absorption and excretion of endogenous and exogenous substances, creates tissue barriers and maintains the homeostasis of the physiological compartments of the body. These activities suggest that it may have an important role in the carcinogenesis of, inter alia, the colon and rectum. First, ABCG2 protects cells against compounds initiating and/or intensifying neoplasia. Dietrich et al. [9] identified elevated concentrations of 2-amino-1-methyl-6-phenylimidazo[4,5-b] pyridine (PhIP), a food-derived colon carcinogen and substrate of ABCG2, in adenomas of ApcMin mice. Downregulation of ABCG2/Abcg2 was found to impair the barrier function of the intestine, thus leading to higher carcinogen concentrations in colorectal adenomas in mice and humans and promoting the adenoma–carcinoma sequence via DNA-bound accumulation of carcinogenic xenobiotics. Moreover, ABCG2 can affect the oral availability and tissue distribution of flavonoids, reducing their beneficial anticancer effect [10].

It is still not fully understood if the downregulation of *ABCG2* expression in colorectal cancer is a cause or a consequence of carcinogenesis. Previously, it was demonstrated that inflammation, a part of colorectal carcinogenesis, decreased the expression of ABC transporters in the intestines in animals. Significantly lower *Abcg2* mRNA levels were noted in the small intestines of adjuvant-induced arthritis rats compared with controls [11]. Englund et al. [12] demonstrated lower *ABCG2* expression in patients with active ulcerative colitis compared with controls, and the level negatively correlated with the *IL-6* mRNA level. Lower ABCG2 staining of the colonic epithelium was noted in inflamed tissues compared to healthy mucosa, and this was associated with disruption of the epithelial F-actin structure. It was also found that inflammation is needed to reduce *ABCG2* mRNA expression because it did not differ between patients in remission and healthy controls. Mossafa et al. [13] demonstrated that proinflammatory cytokines, such as IL-1β, IL-6 and TNF-α, were able to modulate the expression of ABCG2 at transcriptional and post-transcriptional levels in human cervix and gastric cancer cells. Moreover, ABCG2, along with other membrane transporters, is an important component of the intestinal barrier against xenobiotics such as drugs, bacterial toxins or carcinogens such as benzo[a]pyrene conjugates, 17 aflatoxin B1, 25 and PhIP18. Deuring et al. [14] showed that in patients with active inflammatory bowel disease, various inflammatory mediators can block the detoxification function of ABCG2 in intestine mucosa as a consequence of an unfolded protein response. The expression of ABCG2 in the intestine is directly influenced by the expression of the pregnane X receptor PXR, a key regulator in drug metabolism and efflux [15]. Hence, it can be suggested that the downregulation of *ABCG2* expression observed in colorectal cancer can result from inflammation in the bowel mucosa, and that this may represent a preliminary step in reducing its protective potential against cancer-promoting xenobiotics.

The human *ABCG2* gene harbours a variety of polymorphisms and mutations, which may significantly change its expression as well as its substrate binding and transporter activity through improper protein folding or cellular trafficking (reviewed in [16]). To et al. [17] found *ABCG2* mRNA variants that differ in the 3’UTR sequence, and the shorter forms of this sequence do not have a possible binding site for the corresponding microRNA, hsa-miR-519c, thus preventing mRNA degradation and/or repression on protein translation, resulting in transporter overexpression; this is observed in resistant S1MI80 colon cancer cells. Importantly, some studies indicate that *ABCG2* sequence variants may be involved in modulating colorectal cancer risk, as the expression and activity of the transporter in the bowels can differ between individuals, due at least in part to genetic polymorphisms of the *ABCG2* gene. Campa et al. [18] reported an association between colorectal cancer and rs2622621 and rs1481012 *ABCG2*. In addition, Kopp et al. [19] indicated that *ABCB1* rs1045642, *ABCG2* rs2231137 and *IL10* rs3024505 interacting with fibre intake significantly influenced colorectal cancer risk; however, this was contradicted by Andersen [20].

Additionally, ABCG2 is also considered a marker of cancer stem cells (CSCs), a subpopulation of tumour cells with stem cell characteristics; these are believed to be responsible for cancer growth, drug resistance and recurrence. Significantly increased expression of ABCG2 was observed in so-called side population (SP) cells isolated from various human gastrointestinal system cancer cell lines resembling stem cells [21]. Xie et al. [22] found that a fraction of SP cells obtained from colon cancer samples exhibited enhanced *ACBG2* expression compared to non-SP cells.

CSCs are often characterized by the presence of the CD133 cell surface marker. Ma et al. [23] found ABCG2 to be expressed in CD133-positive cancer stem cells from human colorectal tumours. siRNA-mediated knock-down of *ABCG2* expression lowered the self-renewal capacity of the cells and increased the efficiency of chemotherapy-induced apoptosis in colon adenocarcinoma cells and CD133-positive colorectal carcinoma cells. In addition, in SW480 cells, knockdown of *ABCG2* by lentivirus construct inhibits CD133 expression, sphere formation in vitro and tumour formation in vivo [24]. In CSCs, ABGC2 is able to transport compounds important for the growth, division and differentiation of the cells and pump out any harmful endo- and exogenous substances. Krishnamurthy et al. [25] demonstrated ABCG2 maintains CSC survival under hypoxic conditions by reducing the accumulation of protoporphyrins, i.e., toxic heme metabolites.

In addition, Gupta et al. [26] showed that *ABCG2* mRNA and protein levels are decreased several-fold in human colorectal cancer and liver tissue with metastasis from a colonic primary. They postulate that downregulation of ABCG2 may enhance the accumulation of protoporphyrins in the tumour cell, resulting in increased generation of heme, a cofactor for isoform I of nitric oxide synthases, and sustainable production of precancerous nitric oxide during malignancy. It appears that low activity of NOS may be cytostatic or cytotoxic for tumour cells, whereas high activity can have the opposite effect and promote tumour growth.

Some studies have reported the presence of ABCG2 in the nucleus in head and neck squamous cell carcinoma cells [27] and glioblastoma multiforme cells [28]. In lung cancer cells, Liang SC et al. [29] found ABCG2 protein to bind to the E-box of *CDH1* (E-cadherin) promoter inside the nucleus, where it regulates its transcription. Increased expression of ABCG2 causes an increase in E-cadherin and attenuates cell migration in vitro. In contrast, an increased level of ABCG2, and corresponding increase in E-cadherin, may induce circulating cancer cells to colonize at a distant site and form a metastatic tumour. Wang et al. [30] reported strong membranous staining of ABCG2 to be significantly linked with lymph node and distant metastasis, and that cytoplasmatic expression was connected with tumour stage. The researchers postulated that high ABCG2 expression can reduce ROS production and thus confer better OS and DFS, and that the protective role of ABCG2 was specific to the site.

Both the ABCG2 gene and protein expression have aroused considerable interest as potential prognostic factors in various cancers. Both have been shown to be negative prognostic factors associated with a more aggressive phenotype of haematological malignancies such as acute myeloid leukaemia [31] and adult acute lymphoblastic leukaemia [32], and solid tumours such as non-small-cell lung cancer [33], small-cell lung cancer [34], oesophageal squamous cell carcinoma [35,36,37], pancreatic cancer [38], pancreatic ductal adenocarcinoma [39], head and neck squamous cell carcinoma [40] and breast cancer [41]. The expression of ABCG2 protein is correlated with the expression of HER2 in breast cancer, suggesting that ABCG2 is not only a drug-resistance-related transporter but also a potential biomarker predicting the biological behaviour, clinical progression and prognosis of breast cancer [42]. In contrast, loss of ABCG2 protein was related to a worse prognosis and was an independent prognostic factor in patients with moderately or poorly differentiated intrahepatic cholangiocarcinoma [43]. Several studies on the prognostic significance of ABCG2 expression in colorectal cancer have been conducted; however, they have yielded inconsistent results [26,30,44,45,46] due to heterogeneity in the numbers of analysed samples and patient enrolment, stratification schemes, applied treatments and measurement of *ABCG2* gene and ABCG2 protein expression.

Therefore, the present study integrates data regarding *ABCG2* gene and protein expression from publicly available databases. Multiple bioinformatical and biostatistical analyses were conducted, including screening of *ABCG2* expression in a collection of various malignancies, comparing *ABCG2* expression in normal and malignant colon and rectum tissues, evaluating the relationship between the *ABCG2* expression level and clinical features of CRC and prognosis in colorectal cancer, and constructing the functional network of the ABCG2 protein. Furthermore, to validate findings from the in silico analysis, *ABCG2* gene and protein expression was measured in a cohort of colorectal cancer patients to determine their prognostic significance. The study also discusses the significance of the findings from the in silico and wet analysis with regard to those of previous studies.

## 2. Results

### 2.1. In Silico Analysis of OMICS Data Regarding ABCG2 Gene and ABCG2 Protein Expression in Colorectal Cancer

#### 2.1.1. A Decrease in *ABCG2* mRNA Expression Level Is Common in Multiple Carcinomas

**Figure 2:**
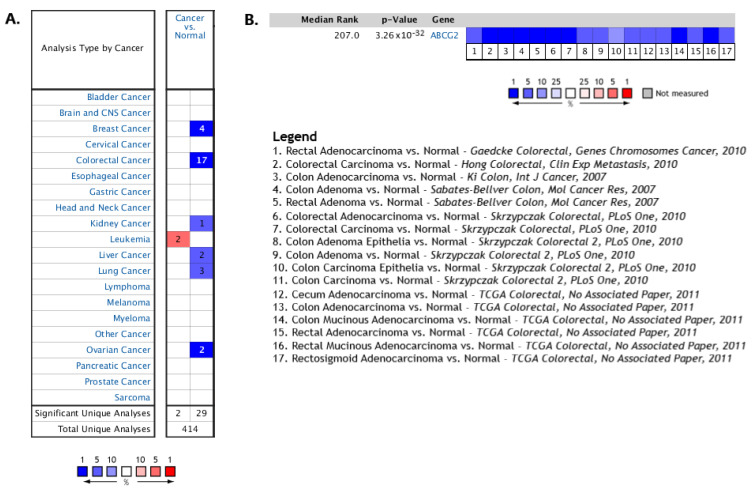
(**A**) The relative *ABCG2* mRNA levels in different types of cancer compared to matched normal tissue in Oncomine. Significantly (*p* < 0.05) increased and decreased levels of *ABCG2* are indicated in red and blue, respectively. The intensity of cell colour is determined by the best gene rank percentile for the analyses within the cell. The number in each cell represents the number of analyses that meet the given thresholds within the analysis and cancer types. (**B**) Comparison of *ABCG2* mRNA expression in colorectal cancer across 17 analyses. The rank given for the gene is the median rank for the gene across each of the analyses. The *p*-value for the gene is its *p*-value for the median-ranked analysis.

First, differences in *ABCG2* expression between cancers of various origins and comparable noncancerous tissue from healthy individuals were assessed via the Oncomine platform. Decreased *ABCG2* mRNA expression (blue) was observed in all except one analysed cancer, i.e., including various breast, ovarian, lung and liver tumours (Figure 2A). In 7 of 12 datasets collected in Oncomine, 17 out of 34 analyses found *ABCG2* to be among 10% of the top underexpressed genes in colorectal tumours (Figure 2B).

**Figure 3:**
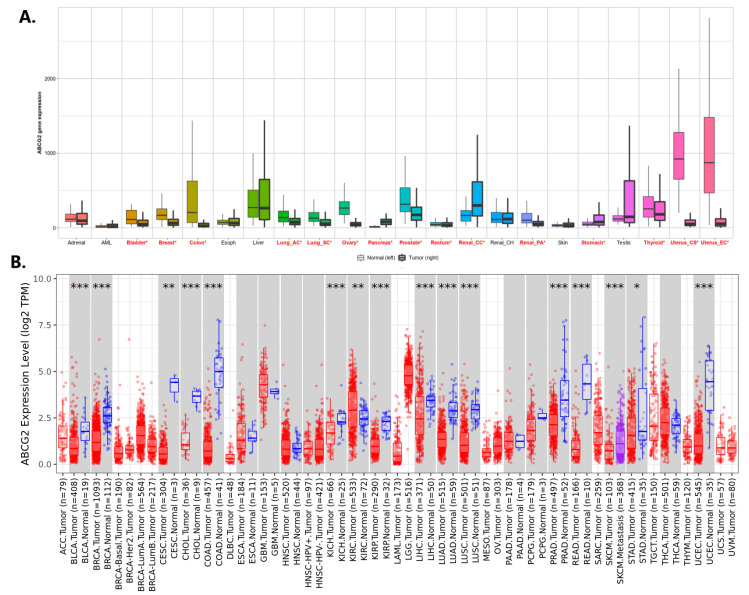
The level of *ABCG2* expression in different cancer types compared to the corresponding normal tissue: (**A**) TNMplot; cancer names where differences with *p* < 0.01 (Mann–Whitney U-test) were detected are typed in red and indicated by asterix. (**B**) TIMER2.0; the statistical significance (*p*-value) computed by the Wilcoxon test is annotated by the number of stars: * < 0.05; ** < 0.01; *** < 0.001; box plots in grey columns indicate cancer types where data for matched normal tissue were available; red and blue box plots indicate tumour and normal samples, respectively.

Comparable results were obtained from the TNMplot and TIMER 2.0 databases, where the majority of cancer types showed significantly decreased *ABCG2* expression (Figure 3A,B), e.g., bladder cancer, breast cancer, lung adenocarcinoma and squamous carcinoma, colon and rectum adenocarcinoma or uterine endometrial cancer. One exception was renal clear cell carcinoma, where significant overexpression of *ABCG2* was confirmed in both datasets.

#### 2.1.2. *ABCG2* Is Underexpressed in Colorectal Cancer in Comparison to Both Adjacent and Unpaired Normal Colorectal Tissue

**Figure 4:**
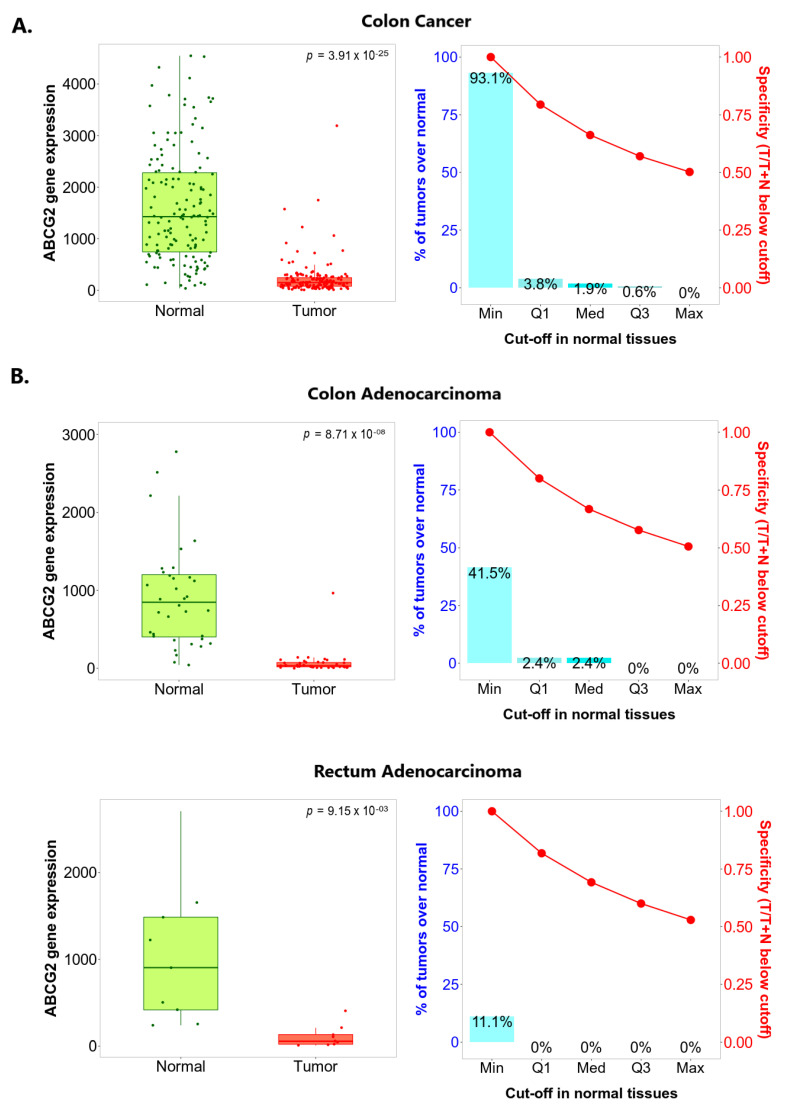
*ABCG2* expression level in paired tumour and adjacent normal tissue according to TNMplot. (**A**) Gene chip data for colon cancer (**B**) RNA-Seq data for colon adenocarcinoma and rectum adenocarcinoma.

**Figure 5:**
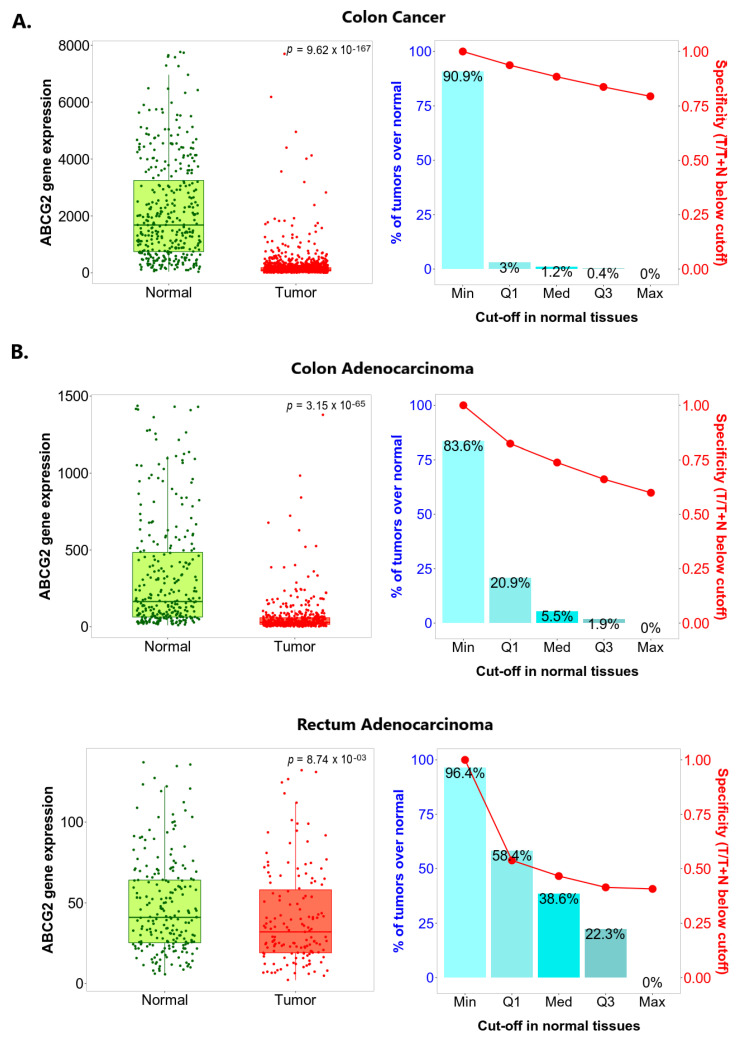
*ABCG2* expression level in non-paired tumour and normal tissue by TNMplot. (**A**) Gene chip data for colon cancer (**B**) RNA-Seq data for colon adenocarcinoma and rectum adenocarcinoma.

To confirm whether *ABCG2* expression changes during carcinogenesis in the colon and rectum, paired colon or rectum cancer tumours and adjacent normal tissue collected from the same patients were compared using the TNMplot platform. As shown in Figure 4, *ABCG2* expression was significantly lower in colon cancer samples than in paired noncancerous tissues (*p* = 3.91 × 10^−25^) indicated by DNA chip data, and significantly lower in both colon and rectum adenocarcinoma samples compared to paired normal tissues, assessed by RNA-seq (*p* = 8.71 × 10^−8^ and *p* = 9.15 × 10^−3^, respectively). Similar differences were noted between tumours and nonadjacent healthy tissue samples (Figure 5). *ABCG2* expression was substantially decreased in cancerous tissue samples compared to non-transformed tissue obtained from the separate subject cohort. Similar results were observed in the case of colon cancer, measured by DNA chip (*p* = 9.62 × 10^−167^), as for colon and rectum adenocarcinomas based on RNA-seq data (*p* = 3.15 × 10^−65^ and *p* = 8.74 × 10^−3^).

The study also evaluated the sensitivity and specificity of *ABCG2* expression as an indicator, with major cutoffs set at the base of the range of *ABCG2* expression in normal samples. The identified sensitivity and specificity are presented in the charts on the right-hand side in Figure 4 and Figure 5. In colon cancer samples, the optimal sensitivity and specificity was found at minimum cutoff with adjacent noncancerous tissue or unpaired normal tissue used as a reference (Figure 4A and Figure 5A). In addition, the best sensitivity (the proportions of tumour samples that show higher expression of the selected gene compared to normal samples at each of the quantile cutoff values) and specificity (calculated by dividing the number of tumour samples with the sum of tumour and normal samples below each given cutoff) were sought. Optimal sensitivity and specificity were also found for the minimum cutoff when colon and rectum adenocarcinoma tumours were analysed against unpaired healthy tissues (Figure 5B). No satisfactory cutoff point was found for the colon and rectum adenocarcinomas when adjacent non-tumour samples were considered as a reference (Figure 4B), probably because of the relatively low number of samples provided for analysis.

#### 2.1.3. *ABCG2* Expression Is Higher in Metastatic Tissues Than in Primary Tumours of Colon Cancer

**Figure 6:**
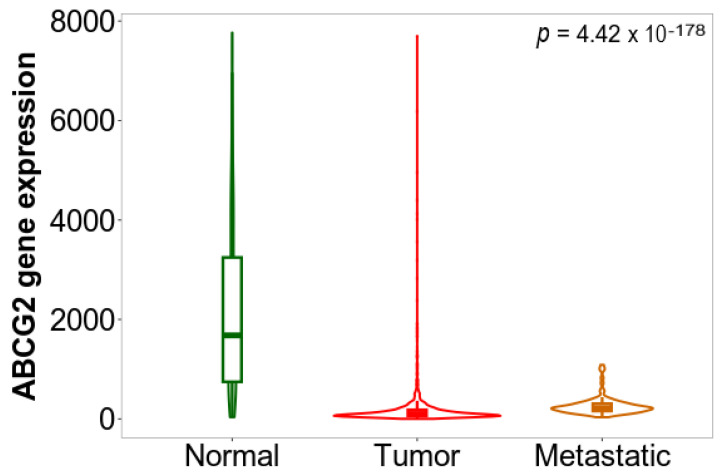
*ABCG2* expression level in normal, tumour and metastatic tissue by TNMplot (gene–chip data).

Additionally, *ABCG2* expression was compared between normal colon tissue, primary tumour and metastatic tissue (Figure 6). A significant difference in the level was found (*p* = 4.42 × 10^−178^). The level was substantially lower in both primary and metastatic tissue than in the normal colon (*p* = 3.77 × 10^−176^ and *p* = 7.22 × 10^−14^, respectively). However, the metastatic tissue showed higher *ABCG2* expression than the primary tumour tissue (*p* = 8.37 × 10^−15^).

#### 2.1.4. ABCG2 Protein Could Be Detected in Colon and Rectum Normal Tissue but Not in Colorectal Cancer

**Figure 7:**
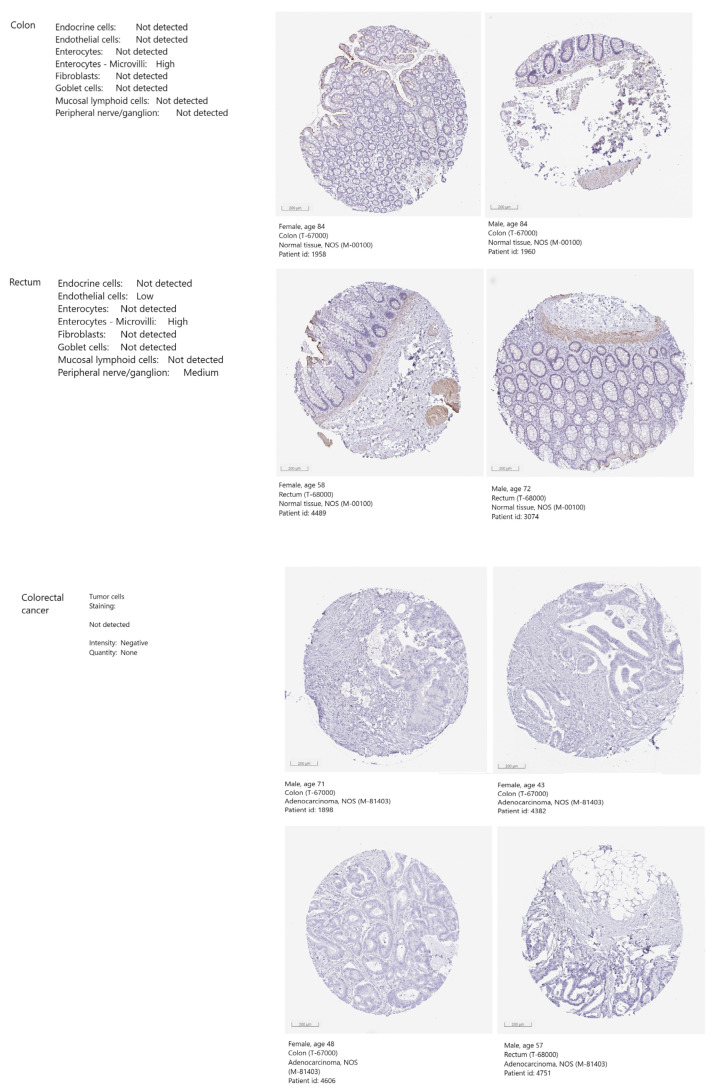
ABCG2 protein expression levels. The images indicate immunohistochemistry staining for normal colon and rectum tissues and colon and rectum adenocarcinomas collected with HPA. Higher antigen content (representing the level of protein expression) and distribution density is indicated by colour rendering: blue = negative; light yellow = weakly positive; brown = moderately positive; dark brown = strongly positive.

As the *ABCG2* mRNA expression was found to be substantially decreased in colorectal cancer, the expression and localization of ABCG2 protein in colorectal cancer and corresponding normal tissues was determined based on the immunohistochemistry staining images collected in the Human Protein Atlas (Figure 7). In both colon and rectum noncancerous tissues, high ABCG2 protein immunostaining was revealed in the microvilli of enterocytes. In the rectum, medium-level immunostaining was detected in peripheral nerve cells and low-level staining in endothelial cells. However, no positive reaction was noted in the cancerous cells of colon or rectum adenocarcinomas.

#### 2.1.5. *ABCG2* Gene Expression Level in Colorectal Cancer Could Be Connected with Methylation Status but Not with DNA Alterations of the *ABCG2* Gene

**Figure 8:**
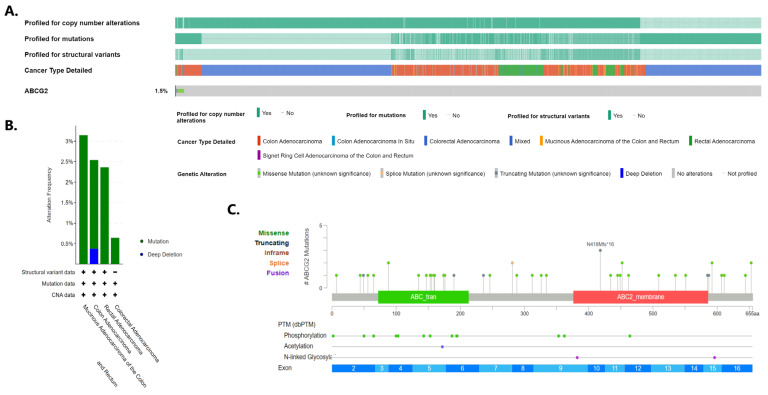
*ABCG2* genomic alterations in colorectal cancer according to cBioPortal. (**A**) Oncoprint of the *ABCG2* in colorectal cancer; (**B**) incidence of different alterations according to colorectal cancer types; (**C**) details of *ABCG2* mutation found in colorectal cancer.

As *ABCG2* was found to be commonly underexpressed in colorectal carcinomas, the mutational and methylation status of the gene was analysed. An Oncoprint was generated by querying 5511 patients/5285 samples from 16 studies using cBioPortal (Figure 8A). In total, *ABCG2* alterations were detected in 1.5% of colorectal cancer patients profiled for mutation, copy number changes and structural variants. With regard to histological subtypes, the highest frequencies of *ABCG2* changes were noted in mucinous adenocarcinoma of the colon and rectum (Figure 8B). Of these, the most commonly detected were mutations, with deep deletion being less common. No amplifications or structural variants were noted. Missense, truncating and splice change mutations were relatively evenly distributed along the gene (Figure 8C).

**Figure 9:**
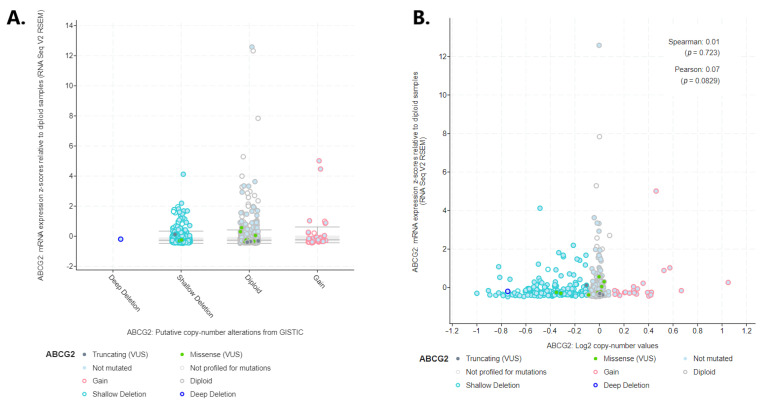
Association between *ABCG2* mRNA expression and (**A**) putative copy-number alteration form GISTIC; (**B**) log2 copy number value (generated by cBioPortal).

The deep deletion mentioned above could be the possible reason for *ABCG2* underexpression. However, no increase in expression was noted when moving from loss of copy number to gain (Figure 9A): the analysis only revealed very weak and insignificant correlation coefficients (R Spearman 0.01, *p* = 0.723; R Pearson 0.07, *p* = 0.829; Figure 9B).

**Figure 10:**
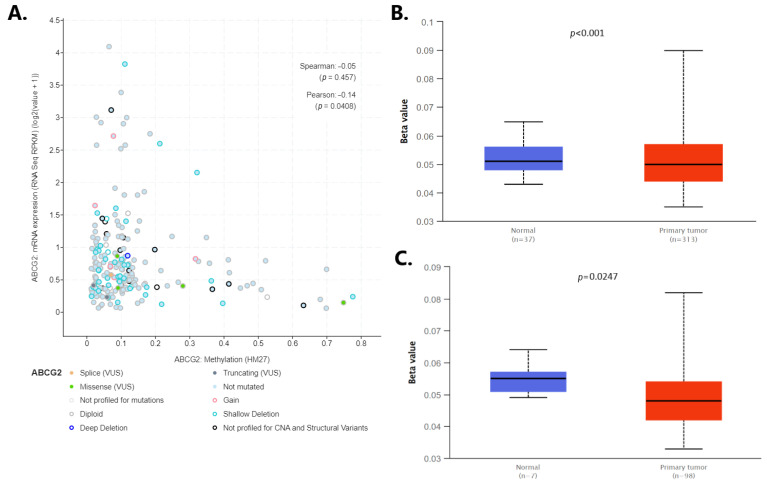
(**A**) Association between mRNA expression and DNA methylation profile of the *ABCG2* gene based on cBioPortal; the promoter methylation level of the *ABCG2* in (**B**) colon adenocarcinoma and (**C**) rectum adenocarcinoma in comparison to normal tissue (by UALCAN).

Changes in gene methylation occur frequently during cancerogenesis, thus influencing the expression of the genes important for transformation, the methylation level of the *ABCG2* gene was inspected in colorectal cancer. According to cBioPortal data (Figure 10A), *ABCG2* expression decreased with increased methylation. However, a statistically significant but weak Person’s correlation coefficient (−0.14, *p* = 0.0408) and insignificant Spearman coefficient (−0.05, *p* = 0.457) were calculated for the association. The methylation level of the *ABCG2* was compared between colon and rectum adenocarcinoma tissue and normal samples using TCGA data provided by UALCAN (Figure 10B,C). While *ABCG2* promoter hypomethylation was noted in both adenocarcinomas and normal samples (beta values range 0.033–0.090), slightly, but significantly, lower methylation was found for both colon (*p* < 0.01) and rectum adenocarcinomas (*p* = 0.0247) in comparison to normal samples. 

**Figure 11:**
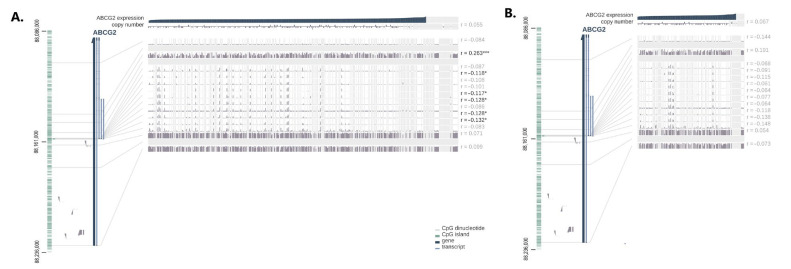
Association between mRNA expression and DNA methylation profile of the *ABCG2* gene by MEXPRESS in (**A**) colon adenocarcinoma (*n* = 557) and (**B**) rectum adenocarcinoma (*n* = 192). On the left side, the *ABCG2* gene together with its transcripts as well as any CpG islands and all the individual CpG dinucleotides were presented. On the right side, each row shows the DNA methylation data for a single probe with Pearson coefficients for the correlation between DNA methylation and gene expression. The promoter probe is highlighted by a black line. Significant coefficients are indicated in black, and *p*-value by using asterisks (*p* ≥ 0.05, * *p* < 0.05, *** *p* < 0.001).

One of the most important factors influencing the regulation of gene expression by DNA methylation is its precise genomic location. Therefore, MEXPRESS visualization was performed of the TCGA data to determine the expression of *the studied gene* and its methylation. For the colon adenocarcinoma (Figure 11A), the level of methylation was negatively correlated with the expression for 5 of 14 probes across the *ABCG2* gene (Pearson correlation coefficients ranging from −0.132 to −0.117). For one probe (cpg location 88147879), a significant positive correlation between the methylation and expression levels was found (Pearson correlation coefficient = 0.263). Contrary to the UALCAN data shown above, no such association was found for the *ABCG2* promoter probe at location 88231061. No significant correlation between methylation and expression was detected for rectum adenocarcinoma (Figure 11B).

#### 2.1.6. *ABCG2* Gene Expression Level in Colorectal Cancer Could Be Connected with Some Clinical Features

**Figure 12:**
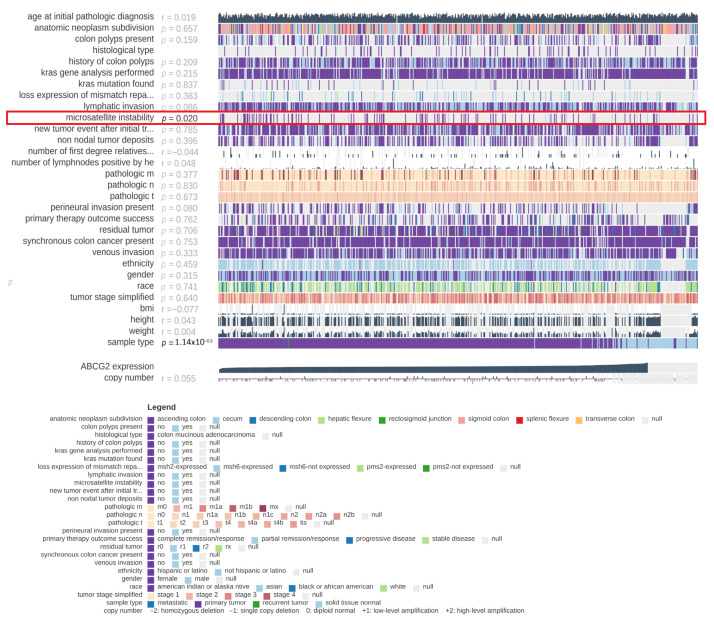
Association between *ABCG2* expression level and selected clinical parameters, generated by MEXPRESS for colon adenocarcinoma samples (*n* = 557). Samples are marked with vertical lines arranged in rows, which correspond to the analysed features. Samples are arranged from left to right of each row by the level of expression of the *ABCG2* gene.

**Figure 13:**
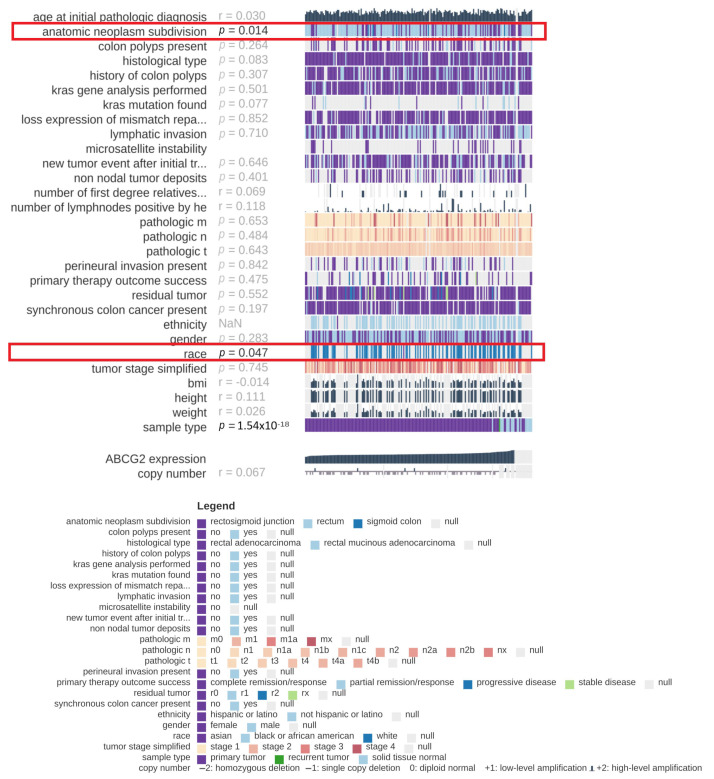
Connection between *ABCG2* expression level and selected clinical parameters. Generated by MEXPRESS for rectum adenocarcinoma samples (*n* = 192). Samples are marked with vertical lines arranged in rows, which correspond to the analysed features. Samples are arranged from left to right of each row by the level of expression of the *ABCG2* gene.

Various clinical factors, e.g., cancer stage, histological type of cancer or tumour localization, influence the clinical course of colorectal cancer. Therefore, the present study evaluates the clinical significance of *ABCG2* gene expression in colon and rectum adenocarcinomas. Using TCGA data and the MEXPRESS online tool, expression was compared to various clinicopathological parameters: localization of the tumour, presence of colon polyps, histological type of cancer, history of colon polyps, presence of *KRAS* mutation, loss of expression of mismatch repair proteins by IHC, microsatellite instability, new tumour event after initial treatment, non-nodal tumour deposits, pathological T, N and M, tumour stage, lymphatic, perineural and venous invasion of cancer, primary therapy outcome success, presence of residual tumour, synchronous colon cancer present, ethnicity, population type, sex and BMI. For colon adenocarcinoma (Figure 12), the only significant association was found between *ABCG2* expression level and microsatellite instability (*p* = 0.020). In rectum adenocarcinoma (Figure 13), *ABCG2* expression was associated with tumour localization (*p* = 0.014) and population type (*p* = 0.047).

**Figure 14:**
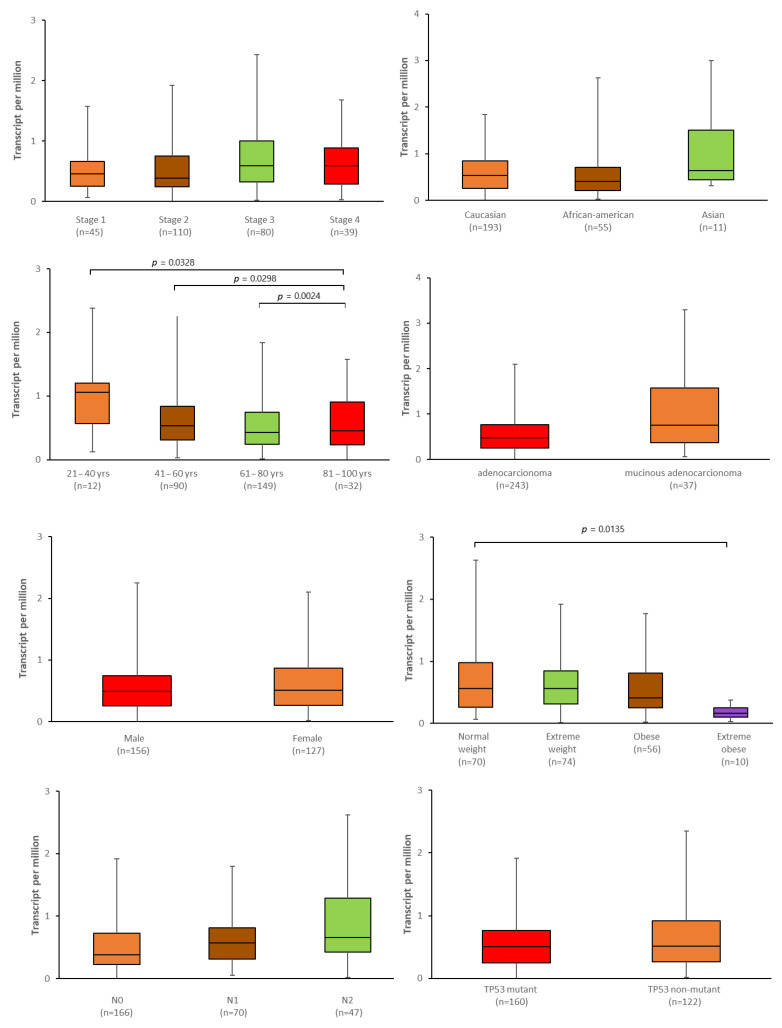
Association between *ABCG2* expression and selected clinical parameters: TNM stage, population affinity, sex, weight, age, cancer histological type, nodal involvement and *TP53* mutation status in colon adenocarcinoma patients and rectum carcinoma patients (UALCAN, modified).

**Figure 15:**
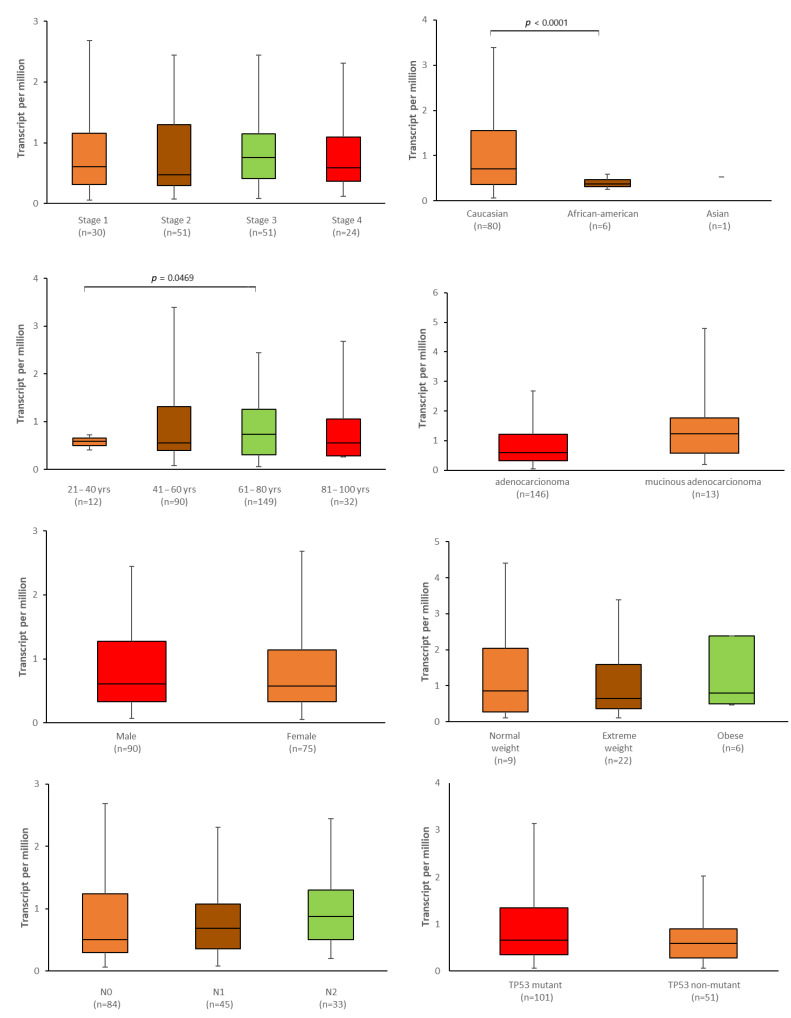
Association between *ABCG2* expression and selected clinical parameters: TNM stage, population affinity, sex, weight, age, cancer histological type, nodal involvement and *TP53* mutation status in rectum carcinoma patients (UALCAN, modified).

Next, to validate and extended the analysis described above, the relationship between the *ABCG2* expression level and selected clinical features was examined with the use of the UALCAN tool (Figure 14 and Figure 15). Although no significant correlation was found between expression and BMI, in the colon adenocarcinoma patients, those of normal weight demonstrated significantly higher expression than extremely obese patients (*p* = 0.0135, Figure 14). In rectal adenocarcinoma patients of Caucasian origin, the expression level of *ABCG2* was significantly higher than in African-American patients (*p* < 0.0001, Figure 15). In both colon and rectum adenocarcinomas, expression was associated with age: the oldest colon adenocarcinoma patients (81–100 years old) showed a significantly lower level of expression than those between 21 and 40 years old (*p* = 0.0328) and between 41 and 60 years old (*p* = 0.0289). They also exhibited a slight but significantly higher expression of *ABCG2* than patients between 61 and 80 years old (*p* = 0.0024). Among rectum adenocarcinoma cases, the youngest patients (21–40 years old) had significantly lower expression levels of the *ABCG2* than patients between 61 and 80 years old (*p* = 0.0496). 

#### 2.1.7. Neither *ABCG2* Gene nor ABCG2 Protein Expression Level Is Related to a Prognosis in Colorectal Cancer

**Figure 16:**
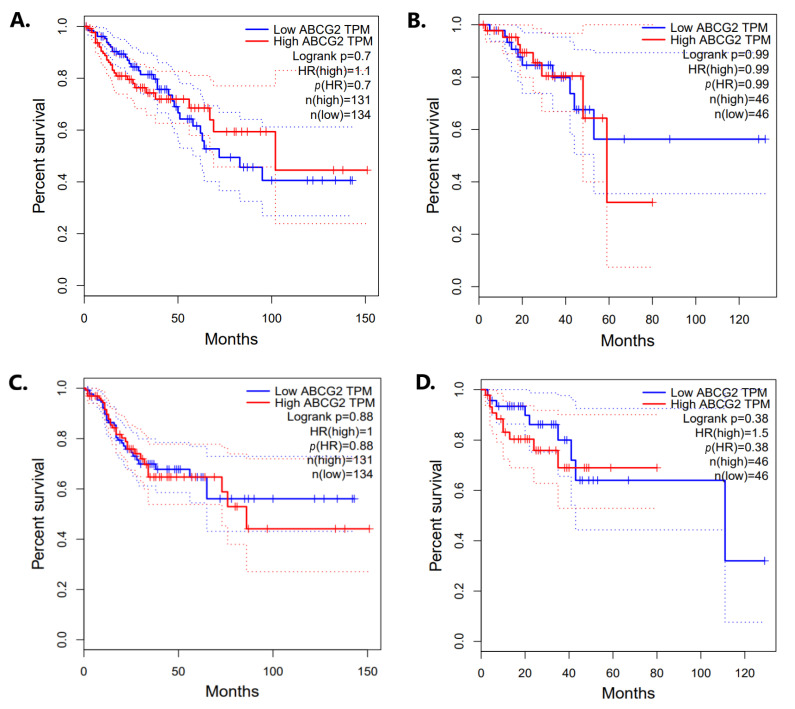
Kaplan–Meier survival curves according to *ABCG2* expression level by GEPIA2: overall survival of COAD (**A**) and READ (**B**) patients; disease-free survival of COAD (**C**) and READ (**D**) patients.

A substantial decrease in *ABCG2* mRNA and protein expression was observed in colorectal cancer, suggesting that it may play an important role in the carcinogenesis process and influence cancer progression. Therefore, GEPIA2 was used to draw Kaplan–Meier plots for the colon and rectum adenocarcinoma patients; these were divided into high- and low-expression *ABCG2* subgroups with median expression as the threshold. Overall survival was not found to differ significantly between the mentioned subgroups for colon (*p* = 0.70) or rectum adenocarcinomas (*p* = 0.99) (Figure 16A,B). Similarly, no significant association was found between expression level and disease-free survival (COAD *p* = 0.88; READ *p* = 0.38) (Figure 16C,D).

**Figure 17:**
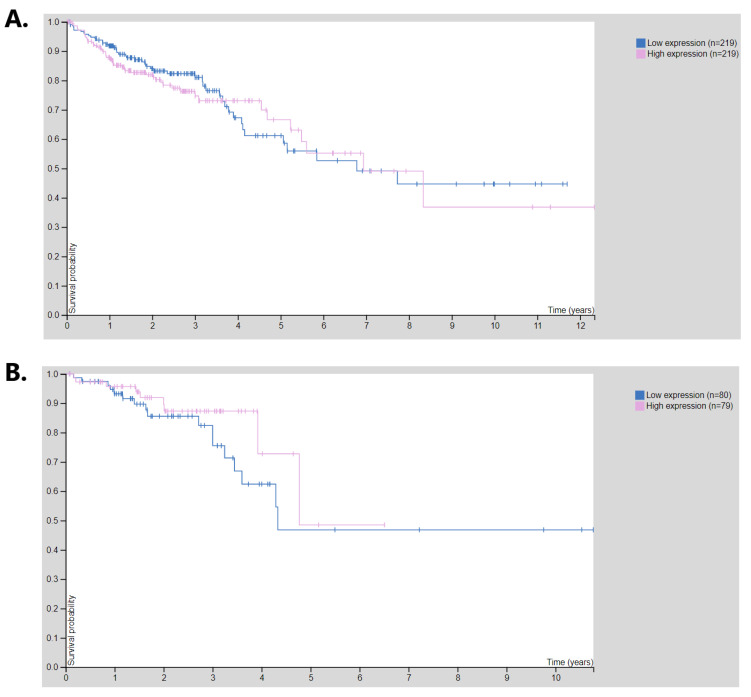
Kaplan–Meier survival curves according to *ABCG2* expression level (Human Protein Atlas: overall survival of COAD patients (*n* = 438) (**A**), READ patients (*n* = 159) (**B**).

Similar results were obtained by Kaplan–Meier curve analysis of *ABCG2* gene expression in the Human Protein Atlas (Figure 17). When cancer patients were divided into two subgroups according to median *ABCG2* expression, no significant difference in overall survival probability was found between the high- and low-expression groups for colon (*p* = 0.51) or rectal adenocarcinomas (*p* = 0.24).

To validate these findings, they were compared with the survival data of colorectal cancer provided by the PrognoScan database (Table 1). Four datasets were retrieved (GSE12945, GSE17536, GSE14333, GSE17537). However, a significant association between *ABCG2* expression and overall survival was found in only one dataset (Cox *p*-value 0.0126, HR 1.45 [1.08–1.94]). In addition, a significant connection between expression and disease-specific survival was noted in only one dataset (*p* = 0.0034, HR 1.58 [1.16–2.14]).

#### 2.1.8. ABCG2 Protein Interacts with Proteins Involved in, e.g., Leukotriene Transport, Endodermal Cell Fate Specification, and Histon Methylation and Ubiquitination

**Figure 18:**
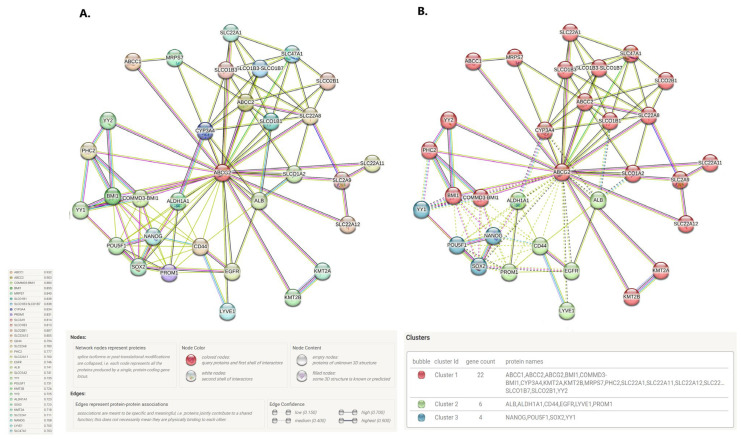
(**A**) The interaction network of the ABCG2 generated by STRING database, (**B**) clustered PPI network of ABCG2; edges of clusters are indicated by dotted lines.

The in silico analysis was completed with the construction of an ABCG2-centered protein–protein interaction network (PPI enrichment *p* < 1.0 × 10^−16^) using the STRING database. Thirty-one interactors were predicted with a confidence score of at least 0.7 (Figure 18A). The top 10 functional partners of the ABCG2 protein were ATP-binding cassette protein C1 (ABCC1, score = 0.932), ATP-binding cassette protein C2 (ABCC2, score = 0.903), COMMD3-BMI1 (annotation not available, score = 0.880), polycomb complex protein BMI-1 (BMI1, score = 0.855), mitochondrial 28S ribosomal protein S7 (MRPS7, score = 0.840), solute carrier organic anion transporter family member 1B1 (SLCO1B1, score = 0.838), solute carrier organic anion transporter family member SLCO1B3-SLCO1B7 (SLCO1B3-SLCO1B7, score = 0.838), cytochrome p450 family 3 subfamily a polypeptide 4 (CYP3A4, score = 0.834), prominin-1 (PROM1, score = 0.831), and solute carrier family 2, facilitated glucose transporter member 9 (SLC2A9, score = 0.814). Within the generated PPI network, three clusters of interaction proteins were identified (Figure 18B). Functional enrichment analysis and gene ontology found the network to be mostly enriched in the following areas: leukotriene transport, xenobiotic transport across the blood–brain barrier, endodermal cell fate specification, histone h3-k4 dimethylation, urate metabolic process, sodium-independent organic anion transport, histone h3-k4 monomethylation, histone h2a-k119 monoubiquitination, bile acid and bile salt transport for biological processes; it was also significantly enriched in PRC1 complex, PcG protein complex, brush border membrane, basolateral plasma membrane and the apical plasma membrane for cellular components.

### 2.2. ABCG2 Gene and ABCG2 Protein Expression in Colorectal Cancer Samples—Wet Validation of the In Silico Analysis Results

#### 2.2.1. ABCG2 Protein Could Be Detected in the Cytoplasm and Membrane of Colorectal Cancer Cells

**Figure 19:**
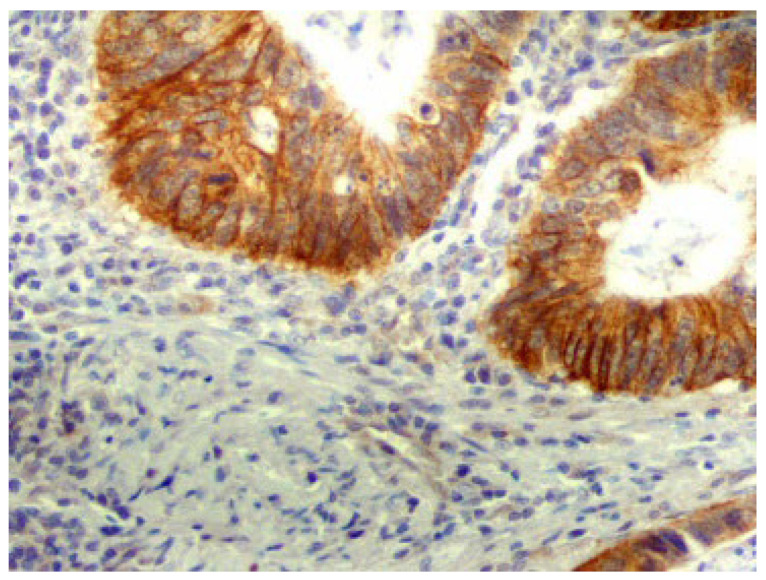
Positive cytoplasmatic and membranous immunohistochemical reaction with antibodies against ABCG2 in colorectal cancer tissue samples (magnification 200×).

Ninety-six samples of colorectal cancer tissue were obtained during the surgical removal of the tumour from patients of the regional oncological centre. Detailed characteristics of the study group are shown in Supplementary Table S1. For immunohistochemical staining, formalin-fixed paraffin-embedded tissue blocks and the primary monoclonal anti-ABCG2 BXP-21 antibody were used. In total, 10 (10.4%) out of 96 tested samples did not express ABCG2 (0% stained cells). A total of 14 samples (14.6%) demonstrated 1–10% cell staining (trace reaction); all these were considered negative for ABCG2 expression. Samples with >10% cell staining (*n* = 72; 75%) were assumed positive for ABCG2 expression. A total of 33 (34.4%) samples had low ABCG2 expression (10–50% stained cells), and 39 (40.6%) samples had high expression (>50% staining). ABCG2 exhibited both cytoplasmic and membranous expression (Figure 19).

#### 2.2.2. *ABCG2* Gene Is Underexpressed in Nearly Two-Thirds of Colorectal Cancer Cases

In the study group, the relative *ABCG2* expression ranged from 0.01 to 731.22 (median 0.68). In 95% of cases, this level ranged from 0.02 to 50. Any outliers were excluded from further analysis (four cases). Nearly two-thirds of cases (*n* = 58, 63.7%) showed underexpression of *ABCG2* to *ACTB*. *ABCG2* expression mainly ranged from 0.51 to 1.0 (27.5%) and from 0.21 to 0.50 (18.7%).

#### 2.2.3. No Association Was Found between *ABCG2* Gene Expression and ABCG2 Protein Levels in Colorectal Cancer Samples

The connection between *ABCG2* gene expression measured using real-time PCR and ABCG2 protein levels assessed by immunohistochemistry was analysed. First, *ABCG2* gene expression was compared in subgroups where ABCG2 protein was recorded or not in the IHC reaction, but no significant connection was found (*p* = 0.381). Second, in cases where the ABCG2 protein was detected, *ABCG2* gene expression was analysed in the high and low ABCG2 protein expression cohorts. Similar to the previous analysis, no significant association was stated (*p* = 0.355).

#### 2.2.4. Neither *ABCG2* Gene Expression Level nor ABCG2 Protein Level Is Connected with Selected Clinicopathological Factors in Colorectal Cancer Samples

Further, the association between ABCG2 protein expression and selected clinicopathological factors was investigated. Subgroups with ABCG2 protein expression were compared with those where expression was absent, and then high and low ABCG2 expression cohorts were compared (Table 2). None of the analysed clinical features was significantly correlated with either the presence of ABCG2 protein or its level, as stated in the IHC reaction.

*ABCG2* gene expression level was also compared with clinicopathological features (Table 3). Similar to the protein, gene expression level was not significantly associated with any considered clinical parameters.

#### 2.2.5. Neither *ABCG2* Gene Expression Level nor ABCG2 Protein Level Is Connected with the Overall Survival Probability of Colorectal Cancer Samples

**Figure 20:**
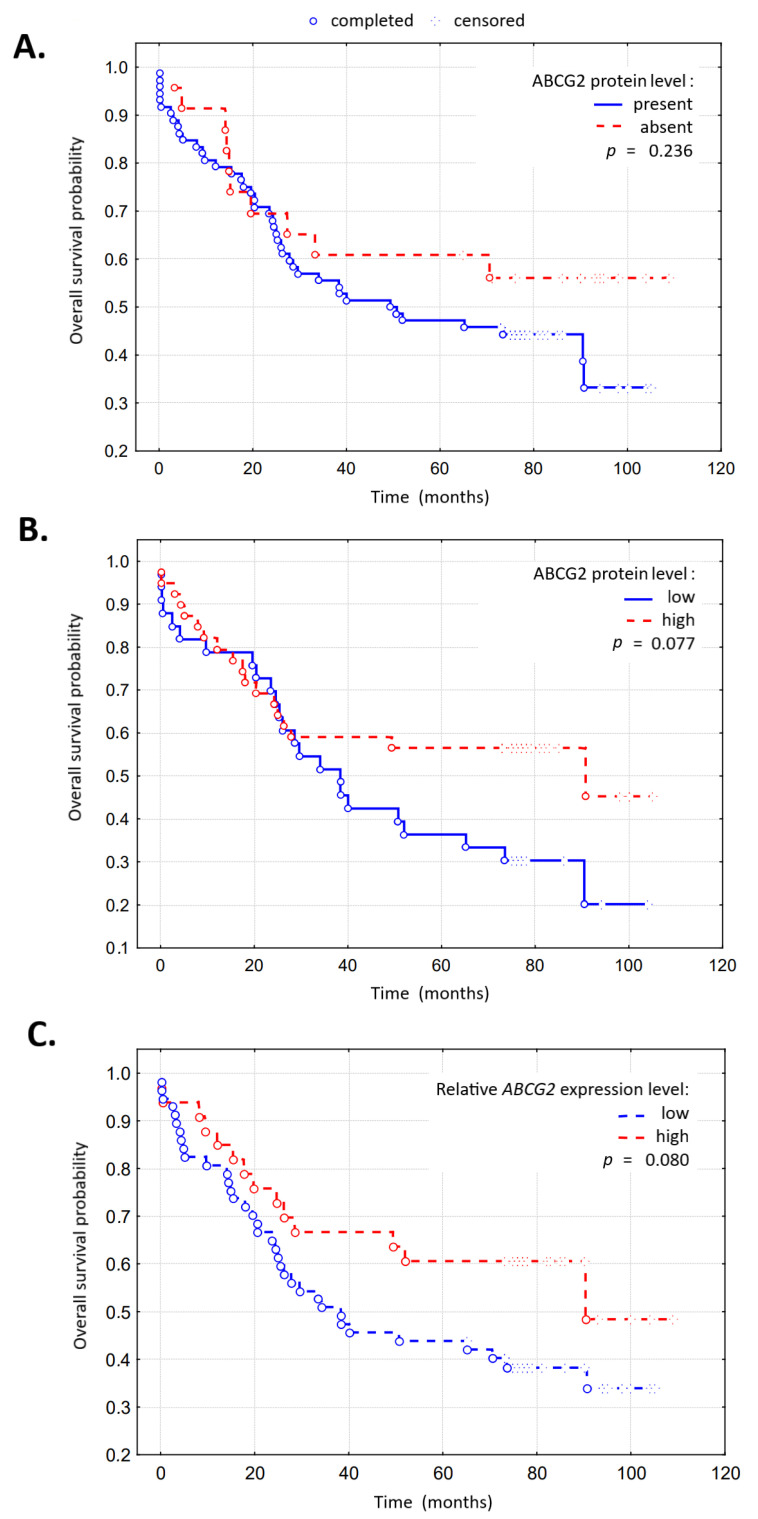
Kaplan–Meier curves for overall survival among colorectal cancer patients with regard to (**A**) the presence of the ABCG2 protein, (**B**) the level of ABCG2 protein and (**C**) *ABCG2* gene expression in tumour tissue samples (*p*-values calculated by log-rank test).

Lastly, Kaplan–Meier curves were prepared to evaluate the influence of ABCG2 protein and gene expression on the survival time of colorectal cancer patients (Figure 20). No significant difference in the survival probability was found between groups with ABCG2 protein present and absent in tumour tissue (*p* = 0.236; Figure 20A). Among the patients with protein expression, overall survival was better in those with high ABCG2 expression, but not significantly (*p* = 0.077; Figure 20B). Similarly, favourable survival was associated with higher levels of *ABCG2* gene expression (above median expression level in the whole group), but, again, the relationship was not statistically significant (*p* = 0.080; Figure 20C).

Significantly better overall survival was connected with a lower depth of tumour invasion (*p* = 0.041), an absence of nodal and distant metastases (*p* = 0.001 and *p* < 0.000, respectively) and the presence of lymphocyte infiltration (*p* = 0.036). The number of deaths and log-rank *p*-values for all analysed parameters are summarized in Table 4.

## 3. Discussion

ABCG2 was first described by Doyle et al. [47] in MCF7/AdVp3000 human breast cancer cells. Because it caused high adriamycin resistance, it was originally named breast cancer resistance protein (BCRP). Since then, the role of *ABCG2* mRNA and protein overexpression in multidrug resistance has been well established in various cancer cell types. This overexpression may serve as a defence against toxic substances such as antitumour drugs.

The present study assessed the *ABCG2* gene expression in a series of diverse malignancies using publicly available big data. Most of the analysed cancer types showed a downregulation of *ABCG2* gene expression. Indeed, decreased gene expression was noted in cancers of distinct tissue origins such as colorectal, bladder, breast, endometrial and lung cancers compared to neighbouring noncancerous tissue. Significantly lower *ABCG2* mRNA level was reported in cancer of 12 organs by Gupta et al. [26]. However, some exceptions were also noted in our analysis; for example, elevated expression was noted in renal clear cell carcinoma. Hence, it appears that *ABCG2* expression level could depend on the cancer type and specificity of tissue origin, and its changes can reflect its role in the carcinogenesis process.

Andersen et al. [48] assessed the role of ABCG2 in the normal–adenoma–carcinoma sequence and found *ABCG2* expression level to be altered in mild/moderate dysplasia, suggesting that this protein is involved in the early steps of carcinogenesis. *ABCG2* mRNA levels were significantly lower in adenomas and carcinomas compared to unaffected tissue from the same individuals and to tissue from healthy; however, the adjacent normal tissue of cancer patients demonstrated higher *ABCG2* expression than the tissue from healthy individuals. The authors suggested that dysfunctions in transport across the epithelial barrier during the transition from mild to moderate dysplasia could enhance the accumulation of carcinogens, thus promoting carcinogenesis in the colon and rectum. Similar results were published by Havlata et al. [49] regarding the primary tumour of colorectal mucosa and paired distant unaffected mucosa. Our present findings strongly support this hypothesis. Our TNMplot analysis revealed that *ABCG2* is underexpressed in both colon and rectum cancer compared to adjacent noncancerous tissue and unpaired noncancerous tissue from healthy individuals. Sensitivity and specificity analysis found that *ABCG2* expression could be a good discriminator between cancerous and adjacent noncancerous tissues in both colon and rectum adenocarcinomas, and it may hence be potentially useful as a colorectal cancer biomarker.

The decrease in *ABGC2* expression level observed in primary colorectal tumours raises the question of whether *ABCG2* is also underexpressed in metastatic tissue. Liu HG et al. [46] reported higher-intensity immunohistochemical ABCG2 protein staining in colorectal cancer cases with positive lymph nodes compared to those with negative nodes. Additionally, ABCG2-positive cells were positioned mainly in the front of carcinomatous tissue or between the carcinomatous and non-carcinomatous margin tissue, which supports the hypothesis that ABCG2 plays an essential role in cancer progression. In our analysis, *ACBG2* expression was significantly higher in colon metastatic tumours than in tumours from the primary location. In contrast, however, Candeil et al. [50] reported that *ABCG2* was highly expressed in the normal colon, and that this expression was dramatically lower in tumoral cells, i.e., colon tumour cells, as well as in untreated hepatic metastases. However, unlike our present findings, they did not detect any significant difference in *ABCG2* expression between cancerous tissue from primary and metastatic locations collected from the same 42 patients, although our present analysis was performed on a much greater number of samples, which were not paired. It could be speculated that while the observed global increase in *ABCG2* expression in metastatic tissue can result from cancer progression, it may also be influenced by the type of therapy. Candeil et al. [50] reported higher *ABCG2* expression in hepatic metastases after irinotecan-based chemotherapy than in irinotecan-naïve metastases.

The frequent deregulation of *ABCG2* expression in various cancers prompted our search for the molecular mechanisms underlying the decrease in *ABCG2* expression in colorectal cancer, namely the sequence changes and methylation of the *ABCG2* gene. In the present study, the data provided by the cBioPortal indicated that *ABCG2* sequence changes are rare events in colorectal cancer. In 5285 analysed colorectal cancer samples, the combined frequency of structural variants, copy number alterations and point mutations was only 1.5%, with the relatively highest frequency in mucinous adenocarcinoma of the colon (about 3%). It is reasonable to assume that the gene expression level should reflect its copy number; however, no increase in *ABCG2* expression level was observed between lower and higher *ABCG2* copy numbers. The low occurrence of the *ACBG2* sequence and copy number changes in colorectal cancer indicates that they are unlikely to be responsible for *ABCG2* underexpression, that another molecular mechanism is responsible for regulating the transcription of this gene in colorectal cancer.

In renal carcinoma cell lines, *ABCG2* gene inactivation was found to be connected with the formation of a repressor complex in the CpG island, which was dependent on DNA methylation [51]. In addition, in multiple myeloma cell lines and ex vivo plasma cells, Turner et al. [52] found the expression of *ABCG2* to be regulated, at least partially, by the methylation of its promoter. Furthermore, differences in the methylation of the promoter upstream region, promoter region and first exon region of the *ABCG2* gene were found between healthy men in China using stool samples [53]. Our present findings indicate significantly lower promoter methylation levels in both colon and rectum adenocarcinomas in comparison to noncancerous tissues; however, only a weak negative correlation was found between *ABCG2* expression and methylation level. Although no significant correlation was observed for the promoter region probe location in either colon or rectum adenocarcinomas, *ABCG2* expression was found to be related to methylation level in other locations in colon adenocarcinoma samples: a negative correlation was noted in five positions and a positive correlation in one. Surprisingly, no such relationship was stated for rectal adenocarcinoma. Hence, it appears that in the *ABCG2* gene, some sites other than the promoter can influence its expression, and that this phenomenon can be restricted to certain localizations of the colorectal tumour.

Studies have indicated that lowered mRNA transcription of *ABCG2* resulted in lowered ABCG2 protein levels. Gupta et al. [26] reported decreased expression of both the ABCG2 mRNA and protein in the luminal surface of colorectal cancer, as well as its liver metastasis, compared to the colorectal epithelium and hepatic tissue in the same patient. Additionally, both *ABCG2* gene expression and ABCG2 protein level were downregulated in colon adenoma with low-grade intraepithelial neoplasia in humans and mice compared to adjacent healthy tissue [9]. Our screening of ABCG2 protein expression in human colon and rectum adenocarcinoma samples deposited in the Human Protein Atlas revealed an absence of ABCG2-specific immunohistochemical staining in the cancerous tissue. However, Maliepaard et al. [54] reported the presence of the protein in healthy colon and rectum enterocytes on the apical membrane of the colon, rectum, jejunum and duodenum. In contrast, higher expression of ABCG2 protein has been reported in colorectal cancer tissue than in non-carcinomatous margin tissues [46]. In addition, elevated expression was noted in about half of the metastatic colorectal tumours studied by Lin P-Ch et al. [55]; however, this group comprised cases ranging from zero to strong expression, with half demonstrating expression in 25–75% of cells. ABCG2 expression was weaker in the normal mucosa than in cancer tissue.

Both the intensity of ABCG2 expression and the proportion of cells expressing it were significantly connected with response to FOLOX [55]. ABCG2 protein expression was observed in 87.1% of cases of CRC tissue from III stage CC [56]. Wang et al. [30] reported ABCG2 protein expression in 96.7% of a large group of colorectal cases, where positivity was considered as more than 10% of tumour cells with an intensity score of at least 1 (weak staining). Assuming 10% positive staining as a minimal cutoff for positivity in our wet analysis, our present findings confirm ABCG2 protein expression in three-quarters of colorectal cancer patients. The discrepancy between the mentioned results may be due to differences in methodology and tissue material.

Our data also indicate both membranous but also cytoplasmatic staining of the cancer cells, which corroborates some previous findings. Gupta et al. [26] reported that ABCG2 was localized at the brush border membrane of normal epithelial cells, and cancer cells showed markedly diminished expression. However, Wang et al. [30] detected the protein in the cytoplasm of over 80% of studied colorectal cancer tissue samples, and in the cell membrane of about two-thirds of cases, where the normal mucosa exhibited strong staining of the apical membrane. Kang et al. [57] reported both cytoplasmatic and membranous ABCG2 expression in over 60% of studied colorectal cancer samples. Both localizations were also detected by Hu J et al. [24] in right-sided colorectal cancer tissues. Palshof et al. [58] reported recently that in addition to the cytoplasm, ABCG2 expression was also present in both the apical/luminal and basolateral membranes of the colorectal cancer cells. It could be speculated that subcellular localization of the ABCG2 can be associated with specific function or loss of function of the transporter during colorectal carcinogenesis. The PI3K/Akt signalling pathway was also found to regulate the translocation of ABCG2 to the plasma membrane and side population phenotype in a mouse model [59]. Altered ABCG2 expression and function could result from EGFR-mediated activation of MAPK cascade [60].

The clinical significance of the *ABCG2* gene and ABCG2 protein expression remains unclear. Some negative findings were published. No association has been found between ABCG2 expression and clinicopathological parameters [56], or between the expression of ABCG2 protein (basolateral, apical or cytoplasmatic) and age, sex, WHO performance status, location of the primary tumour, number of metastatic sites or liver or lung metastases in colorectal cancer [58]. Gupta et al. [26] indicated no significant correlation between *ABCG2* mRNA level and age, sex, population type, grade, stage or localization, and Halavata et al. [49] reported no connection between *ABCG2* expression with the grade, primary localization, T, N, M, age at diagnosis or sex. No difference in *ABCG2* gene expression was found between mucinous and nonmucinous colorectal cancer [44].

In contrast, some significant associations have been reported. In one study, neither cytoplasmatic nor membranous expression of ABCG2 protein was found to be connected with age, sex, tumour site or TNM stage [57]; however, higher expression in both localizations was linked with more pathologically differentiated lesions. No connection was noted between ABCG2 protein positivity and age, sex, tumour size or tumour shape, but higher TNM stage, poor differentiation and positive lymphovascular invasion were found to be associated with greater ABCG2 expression [24]. Additionally, the ABCG2 protein was detected more frequently in cases without perineural invasion [57].

Considering these conflicting reports, the present study analysed the relationships between various clinicopathological factors and *ABCG2* expression using publicly available TCGA datasets. From the large number of factors analysed, only a few were found to be connected with *ABCG2* expression, and different associations were observed for distinct anatomic cancer locations. *ABCG2* expression was significantly associated with microsatellite instability and patient weight in colon carcinoma, and with anatomical organ subdivision, patient age and population type in rectum adenocarcinoma. Unfortunately, our validation analysis in colorectal cancer patient cohort did not identify any significant association between *ABCG2* expression, at the mRNA or protein level, and clinicopathological parameters.

As our present findings, and some previous studies, found ABCG2 gene and/or protein expression to be associated with colorectal cancer clinical parameters, the present study also attempted to translate these associations into patient survival. The existing literature was again divided. High ABCG2 protein expression was associated with poor prognosis [30,45,46]. Hu, J. et al. [24] showed that ABCG2 positive staining of right-sided colorectal cancer was associated with a decreased 5-year survival rate, whereas the opposite was reported by Gupta et al. [26]. Moreover, Palshof et al. [58] reported no association of ABCG2 protein with RFS or OS. Silvestris et al. [61] did not find any connection between ABCG2 expression and patient survival in metastatic colorectal cancer patients.

In our in silico analysis, no significant connection was found between *ABCG2* expression and disease-free or overall survival in either colon or rectum adenocarcinoma patients. Moreover, overall survival did not correlate with ABCG2 protein or *ABCG2* gene expression in our colorectal cancer cohort. Some reports have indicated that cell localization of ABCG2 could determine its influence on survival. Kang et al. [57] found high expression of membranous ABCG2 to be associated with better overall and disease-specific survival; however, no such association was detected for cytoplasmatic ABCG2. After stratification of patients according to TNM stage, only stages II and III demonstrated an association with OS and DFS, and high membranous expression was an independent prognostic factor of OS and DFS. This may be due to the transportation activity of the epithelium and the protective function of membranous ABCG2, which could influence survival time.

Kim et al. [62] reported that ABCG2 protein expression was associated with favourable disease-free survival but not overall survival. However, it was not an independent indicator of OS and DFS. In line with these findings, Han et al. [56] noted that ABCG2 protein positivity was connected with prolonged overall and disease-free survival of CIII stage patients treated postoperatively with FOLFOX-4 chemotherapy. Positivity for ABCG2 was an independent prognostic positive indicator of OS, and ABCG2 negativity was connected with an almost three-times-higher risk of death. In contrast, Wang et al. [30] showed that strong membranous expression of ABCG2 correlated with the lymph node and distant metastasis and Dukes stage, while the cytoplasmic expression was connected with tumour stage only. However, strong membranous expression was linked with shortened survival, but cytoplasmatic expression was not. This contradicts the suggestion that ABCG2 protects cancer cells from harmful substances and prolongs the life of cancer cells; however, this could be connected with some other properties of ABCG2. Giampieri et al. [45] correlated a panel of stemness markers with clinical outcome in resected stage II and III colon cancer patients; *ABCG2* was found to be among those genes with a higher “weight” in determining different prognoses: patients with higher expression of ABCG2 have a worse prognosis (time to relapse).

In the absence of a clear connection with clinical parameters and prognosis, significant alterations in *ABCG2* gene and protein expression in colorectal cancer may suggest that ABCG2 has a complex role in tumorigenesis that is not directly or solely related to its transport function. It is possible that this may be a result of its interaction with other cellular components. Therefore, in the last part of the in silico analysis, a functional protein–protein interaction network was built to identify the molecular partners of ABCG2 that could mediate or enhance the carcinogenic function of the transporter. STRING analysis revealed that ABCG2 collaborates with proteins grouped into three main clusters. The largest cluster comprised various SLC and ABC transporters, which are critical for the absorption, distribution, metabolism and elimination of different drugs and endo-/exogenous toxins. Some of these proteins are involved in the regulation of the physiological molecular signalling network between the intestines, liver and kidneys (ABCC2, SLC22A1, SLC22A8, SLCO1B1) [63]. In addition, the same elements of the ABCG2 network are considered together as important predictive factors during assessing the effectiveness of single drugs (e.g., irinotecan, 5-fluorouracil) or full therapeutic regimens (e.g., FOLFIRI, FOLFOX) [64].

The second network cluster contains e.a. molecules important for cell interaction and signalling. CD44 is a major cell surface receptor for hyaluronic acid (HA), and its expression enhances CSC aggregation. Additionally, the level of phosphorylated transmembrane tyrosine kinases (e.g., ERBB2) and its interactions with other signalling factors in colon cancer cell lines may depend on endogenous HA and CD44 interaction [65]. In other types of cancer, HA-CD44 binding also plays a role in triggering signals from later receptors in the tyrosine kinase family (such as EGFR), leading to PI3K/Akt or MAPK pathway activation [66]. Monoclonal antibodies against EGFR are commonly used in the treatment of metastatic colorectal cancer, and some of these agents synergistically inhibit both EGFR phosphorylation and ABCG2 drug efflux activity [67,68,69]. EGFR was also found to exert a post-transcriptional effect on ABCG2 expression via the PI3K/AKT and RAS/RAF/MEK/ERK signalling pathways [63,64,65]. Bleau et al. [70] reported that PTEN/PI3K/Akt signalling regulates ABCG2 activity in mouse and human gliomas. Mutual regulation has also been confirmed in other studies, indicating that increased expression of both *ABCG2* and *EGFR* (metastatic marker) is positively correlated with resistance to anoikis [71] and metastatic potential [72] in the colorectal cancer cell population.

The third cluster of the built PPI network is well represented by *transcriptional factors* e.g., such as SOX2, NANOG and POU5F1/OCT4, which are *considered cancer stem cell (CSC) markers*. Some phenotypic markers (e.g., CD44 or *BMI-1*) and other stemness-related factors (e.g., ALDH1) [73] can also be found in the other clusters of the network. CSCs often demonstrate resistance against chemotherapeutics due to high expression of ABC transporter genes. Similarly, cancer stem-like side population cells, which may be identified or mediated, among others, by ABCG2 transporter activity, show an increased tendency to proliferation, colony formation, invasiveness and multipotent differentiation; in addition, they may be more tumorigenic and resistant to chemotherapeutic drugs. The side population of colon cancer cell line SW480 exhibits high *ABCG2* mRNA and transporter expression, accompanied by high *CD44* mRNA and protein levels, regarded as a key marker of solid tumour CSCs [74]. Another important marker of colorectal CSCs is BMI-1. Both the CD44v6 isoform and BMI-1 alone identify CSCs [75].

Another group of CSC markers comprises pluripotency transcription factors. Spheroid culture from the HT-29 cell line, a more realistic colorectal cancer in vitro model, shows significantly higher expression of *ABCG2*, *NANOG, SOX2* and *POU5F1* compared to 2D cell culture conditions [76]. It has been found that the E1A isoform of the *ABCG2* transcript, whose expression in human embryonic stem cell lines correlates with the level of *POU5F1* and NANOG, may be responsible for the increased level of total *ABCG2* mRNA in CSCs [77]. Moreover, in mouse embryonic stem cell lines, transfection-mediated inhibition of *ABCG2* downregulates the expression of Nanog, possibly leading to a subsequent reduction in its downstream target POU5F1/Oct-4. This phenomenon may be mediated by changes in the nuclear level of TP53; it could also contribute to cell arrest in the G1 phase of the cell cycle, and thus the removal of such cells from the self-renewal pool [78]. The activity of ALDH1, a member of the second network cluster, is another characteristic feature of both normal and cancer stem cells [79]. As previously mentioned, CSCs are not only small, pluripotent cells that can enter a reversible cell cycle arrest, but they are also the most drug-resistant subpopulation of tumour cells. They are certainly promoted by the mutual co-expression of *ABCG2*, *ALDH1A1* and *CYP3A4* (a member of the first network cluster) observed, e.g., in CSCs of the COLO 205 line [80]. Increased ALDH1A1, a key ALDH isozyme in stem cells, decreases the reactive oxygen species (ROS) level, prevents apoptosis, provides radioresistant properties and has a protective function against cytotoxic drugs [81]. Taken together, the string network brings new information on potentially important partners of ABCG2, which may explain its role in carcinogenesis. However, because the network of interactions is created mainly based on the co-expression of particular genes, further multifaceted research is required to understand the nature of the mechanism of the association between network members.

## 4. Materials and Methods

### 4.1. In Silico Analysis

#### 4.1.1. *ABCG2* Gene Expression and ABCG2 Protein Level Analysis

##### Oncomine

*ABCG2* mRNA expression level was analysed in a variety of human cancers using the Oncomine database [82] (https://www.oncomine.org, accessed on 1 January 2022). The following threshold settings were used: gene ranking of the top 10%, change ≥ 2, *p*-value ≤ 1 × 10^−4^. All statistical methods and statistical values were obtained directly from the mentioned database.

##### TIMER2.0

The differential expression of the *ABCG2* gene in TCGA tumours was compared between tumour and adjacent normal tissues using the Gene_DE module of the Tumor Immune Estimation Resource 2.0 [83] (http://timer.cistrome.org, accessed on 1 January 2022). The distributions of gene expression levels are displayed using box plots. The statistical significance (*p*-value) was computed by the Wilcoxon test.

##### TMNplot

The TMNplot web tool [84] (https://tnmplot.com, accessed on 1 January 2022) was used (1) to display pan-cancer changes in *ABCG2* expression based on RNA-seq data from TCGA, genotype-tissue expression (GTEX), therapeutically applicable research to generate effective treatment (TARGET) (significant differences are given in red and marked with an asterisk), (2) to compare *ABCG2* expression level in colorectal cancer and non-tumour colon and rectum tissues based on RNA-seq and DNA chip data, and (3) to compare the *ABCG2* expression in normal colon tissue, tumours and metastatic tissue of colon cancer based on DNA chip data. The normal and tumour samples were compared by the Mann–Whitney U-test, and matched tissues with adjacent samples were compared using the Wilcoxon test. Normal–tumorous–metastatic tissue comparison was done using the Kruskal–Wallis test and Dunn’s test.

##### Human Protein Atlas

Example images of immunohistochemistry staining of normal colon and rectum tissues, as well as the colon and rectum adenocarcinomas, were obtained from the Human Protein Atlas [85] (https://www.proteinatlas.org/, accessed on 29 March 2023). The methods of obtaining and analysing the available data are described in detail on the HPA websites: https://www.proteinatlas.org/humanproteome/tissue/method (accessed on 22 May 2023); https://www.proteinatlas.org/humanproteome/pathology/method#the_pathology_section___methods_summary (accessed on 22 May 2023).

#### 4.1.2. Analysis of DNA Alteration and Methylation of the *ABCG2* Gene

##### cBioPortal

The genomic characteristics of *ABCG2* in colorectal cancers were analysed using the cBioPortal for Cancer Genomics [86] (v3.7.28; http://www.cbioportal.org, accessed on 4 February 2023). The query comprised 5511 patients/5285 samples from 16 studies. The incidence of different alterations of the studied gene was assessed in colorectal cancer cases and, specifically, in colorectal cancer histological types. Additionally, the association between *ABCG2* mRNA expression level and copy number alteration and between the mRNA level of the gene and methylation beta-value (HM27) was analysed. Spearman’s and Pearson’s correlation coefficients were calculated.

##### UALCAN

The association between *ABCG2* expression and *ABCG2* promoter methylation in the colon and rectum adenocarcinomas was determined using Ualcan TCGA data available on the UALCAN portal [87] (http://ualcan.path.uab.edu/index.html, accessed on 1 January 2022). The results were presented in box-whisker plots with the minimum, q1, median, q3 and maximum values. The presented beta-value is the ratio of the methylated probe intensity and the overall intensity (sum of methylated and unmethylated probe intensities). The significance of the difference was estimated by Student’s *t*-test considering unequal variance.

##### MEXPRESS

The relationship between TCGA expression and DNA methylation data for the *ABCG2* gene was determined using the MEXPRESS visualization tool (https://www.mexpress.be/, accessed on 22 May 2023) [88]. Pearson correlation coefficients and Benjamini-Hochberg-adjusted *p*-values were calculated for the comparison between the methylation level for each specific probe and the *ABCG2* expression level.

#### 4.1.3. Analysis of Connection of *ABCG2* and Clinicopathological Features

##### MEXPRESS

The connection between TCGA expression and clinical data for the *ABCG2* gene was determined with the MEXPRESS visualization tool (https://www.mexpress.be/, accessed on 22 May 2023) [88]. Pairs of continuous variables were compared using Pearson’s correlation coefficient, while continuous and categorical variables were compared using a *t*-test or ANOVA. Benjamini-Hochberg-adjusted *p*-values are provided.

##### UALCAN

The association between *ABCG2* expression and selected clinical features in colon adenocarcinoma and rectal adenocarcinoma was validated using TCGA data available on the UALCAN portal [87] (http://ualcan.path.uab.edu/index.html, accessed on 29 March 2023). The results were presented in box-whisker plots with the minimum, q1, median, q3 and maximum values. The significance of the difference was estimated by Student’s *t*-test considering unequal variance.

#### 4.1.4. Prognosis and Survival Analysis

##### GEPIA2

The GEPIA2 [89] (http://gepia2.cancer-pku.cn/#survival, accessed on 29 March 2023) was applied to evaluate the prognostic value of *ABCG2* expression for overall survival and disease-free survival in colon and rectal adenocarcinomas. Kaplan–Meier plots were drawn using “ABCG2” as an input query, and patients were split by median; the hazard ratio was calculated based on the Cox PH model.

##### Human Protein Atlas

The prognostic value of ABCG2 protein expression regarding overall survival in colon adenocarcinoma and rectal adenocarcinoma was evaluated using data from the Human Protein Atlas [85] (https://www.proteinatlas.org/, accessed on 29 March 2023). Patients were split by median *ABCG2* expression, i.e., the median FPKM value calculated from the gene expression (FPKM) data from all patients in the dataset. Log-rank *p*-values for Kaplan–Meier plots were provided.

##### PrognoScan

The association between *ABCG2* expression level and overall or relapse-free survival in colon and rectal adenocarcinomas was determined using PrognoScan [90] (http://dna00.bio.kyutech.ac.jp/PrognoScan/index.html, accessed on 1 January 2022). Cox *p*-values and hazard ratios with a 95% confidence interval were calculated according to *ABCG2* mRNA level (high vs. low).

#### 4.1.5. Protein–Protein Interaction Analysis

##### STRING

A protein–protein interaction (PPI) network querying the protein “ABCG2” and organism “Homo sapiens” was created using the STRING database [91] (https://string-db.org/, accessed on 1 January 2023). The main parameters were set as follows: the minimum required interaction score was 0.7 and no more than 50 interactors to show. K-means clustering of the generated PPI network was performed with a pre-set of three clusters.

### 4.2. ABCG2 Gene Expression and ABCG2 Protein Analysis in Colorectal Cancer Samples

#### 4.2.1. Patients and Tissue Samples

A total of 96 patients of the Oncological Center of Łódź, Poland, with colorectal carcinomas were enrolled in the study. Cancer tissue samples were obtained from the patients during the surgical removal of the tumour. Detailed characteristics of the study group are shown in Supplementary Table S1.

Tumour tissues intended for molecular analysis were frozen immediately after collection in liquid nitrogen and stored at −80 °C until analysis. In addition, tissues for immunohistochemical analysis were fixed in 10% PBS-buffered formalin and embedded in paraffin blocks. Histological diagnosis and clinical staging were performed for each patient. All experiments were carried out with the local ethical committee approval (RNN/83/20/KE) and the patient’s informed consent.

#### 4.2.2. ABCG2 Protein Level Analysis by Immunohistochemistry

Briefly, 4 μm sections of formalin-fixed, paraffin-embedded tissue were placed on SuperFrost Plus slides (Menzel-Glaser, Braunschweig, Germany). These were deparaffinized in xylenes and rehydrated through graded alcohol. Then, the sections were microwaved in 0.01 M sodium citrate buffer, pH 6.0, twice for 10 min at 360 W for epitope retrieval. The slides were then washed with TRIS buffered saline, pH 7.4, and incubated for 1 h at room temperature with the primary monoclonal antibody anti-ABCG2 (clone BXP-21, 1:25 dilution, Chemicon International, Temecula, CA, USA) and processed with EnVision+ (DAKO, Glostrup, Denmark) system. Sections were counterstained with haematoxylin, dehydrated with ethanol and cleared in xylene. Negative controls were obtained by omitting the application of the monoclonal antibody. Expression was assessed by counting the positive cell reactions. Depending on the number of cells, the cases were divided into four classes: 0% of cells stained—no expression, 1–10% of cells stained—trace expression, 11–50% of cells stained—low expression, 51–100% of cells stained—high expression; the cases with more than 10% of cells with a positive reaction were considered positive (adopted after [92]).

#### 4.2.3. RNA Isolation and cDNA Synthesis

Total RNA was isolated from frozen tissue sections (50–100 mg) with TRI Reagent (Sigma-Aldrich, St. Louis, MO, USA) according to the manufacturer’s instructions. The obtained RNA was stored at −80 °C until further analysis. Reverse transcription was performed according to the Enhanced Avian protocol HS RT-PCR Kit, Two-Step Reaction (Sigma-Aldrich, USA) using 400 ng of total RNA. The obtained cDNA was stored at −20 °C until further analyses.

#### 4.2.4. Real-Time PCR Reaction

The reaction mixture consisted of 12.5 µL of the mixture SYBR^®^Green JumpStart™ Taq ReadyMix™ (Sigma-Aldrich, USA), 0.5 µL of each primer (final concentration: 0.2 µM), 9 µL of sterile, nuclease-free water and 2.5 µL of previously prepared cDNA. Together, negative control samples were also reacted with the test samples, which contained all components of the reaction mixture as the test samples except cDNA. They were replaced with 2.5 µL of sterile, nuclease-free water. All reactions were made in triplicate. After each reaction, the melting curve of the obtained products was determined. The reaction was carried out in a MiniOpticon™ System thermocycler (Bio-Rad Laboratories, Hercules, CA, USA). The primer sequences and reaction conditions are as follows: *ABCG2* Forward 5′CCT TAG TTA TGT TAT CTT TGT G3′; *ABCG2* Reverse 5′GTG GGG CGC CCC AGG CAC CA3′; *ACTB* Forward 5′GTG GGG CGC CCC AGG CAC CA3′, *ACTB* Reverse 5′CTC CTT AAT GTC ACG CAC GAT TTC3′; 35 cycles: 94 °C-15 s; 59 °C-45 s; 72 °C-45 s. The relative expression of the *ABCG2* gene was determined according to Pfaffl [93].

#### 4.2.5. Statistical Analysis

Statistical analysis was performed using Dell Statistica version 13, Dell Inc. (2016). To investigate the relationship between qualitative or quantitative characteristics in nominal scales, the χ^2^ test, χ^2^ test with Yates’ correction, and V^2^ tests were used. The normality of the distribution of the continuous variables was determined using the Shapiro–Wilk W test. The nonparametric Mann–Whitney U-test was used to determine the significance of differences in continuous variables between the two groups. Overall survival analysis (time between surgery and death) was performed using Kaplan–Meier curves. Observed differences in survival probabilities were tested by the test log rank (univariate analysis). In all analyses, statistical significance was assumed for *p* < 0.05.

## 5. Conclusions

The findings from the in silico analysis and wet experiments indicate that *ABCG2* gene expression is commonly deregulated in cancerogenesis, and a decrease in the expression of the gene is a general feature of colorectal cancer cells. This downregulation is not driven by *ABCG2* gene sequence or copy number changes, but it can be connected with the methylation level of some sites in the gene. The role of ABCG2 in colorectal cancerogenesis could be linked with the transport function of the protein, but it could also indicate its participation some signalling pathways or protein interactors, which may determine the role of ABCG2 in cancer cell self-renewal and behaviour. As these ABCG2 partners could also influence the clinical significance of ABCG2, simple analyses of *ABCG2* or ABCG2 expression alone cannot yield clear conclusions. These interactions require further in-depth research to reveal the significance of *ABCG2* and its protein expression in colorectal cancer.

## Figures and Tables

**Table 1:** *ABCG2* expression and survival data of colorectal cancer patients using the PrognoScan database.

Dataset	End-Point *	Probe ID	N	Cut-Point	Cox *p*-Value	HR [95% CI]
GSE12945	DFS	209735_at	51	0.75	0.3906	0.53 [0.13–2.24]
GSE12945	OS	209735_at	62	0.29	0.7889	1.07 [0.66–1.72]
GSE17536	OS	209735_at	177	0.87	0.0126	1.45 [1.08–1.94]
GSE17536	DFS	209735_at	145	0.90	0.1210	1.44 [0.91–2.28]
GSE17536	DSS	209735_at	177	0.87	0.0034	1.58 [1.16–2.14]
GSE14333	DFS	209735_at	226	0.82	0.1959	1.23 [0.90–1.69]
GSE17537	DFS	209735_at	55	0.15	0.1856	1.32 [0.87–2.00]
GSE17537	DSS	209735_at	49	0.82	0.6398	1.17 [0.61–2.21]
GSE17537	OS	209735_at	55	0.76	0.3611	1.22 [0.80–1.86]

* OS—overall survival, DSS—disease-specific survival, RFS—relapse-free survival.

**Table 2:** The relationship between ABCG2 protein level and selected clinicopathological factors.

Feature	ABCG2 Protein Level					
	Absent	Present	*p*-Value	Low	High	*p*-Value
Age						
up to 60 y	11	34	0.906 *	16	18	0.844 *
over 60 y	13	38		17	21	
Sex						
female	13	37	0.814 *	17	20	0.984 *
male	11	35		16	19	
Tumour localization						
rectum	8	27	0.939 ^&^	13	14	0.825 *
colon	16	44		20	24	
Histological type						
adenocarcinoma tubulare	19	64	0.389 ^#^	31	33	0.380 ^#^
adenocarcinoma mucinosum	5	8		2	6	
Histological grade						
G1 or G2	15	52	0.898 ^&^	22	30	0.336 ^&^
G3	9	20		11	19	
Depth of tumour invasion						
pT1 or pT2	5	24	0.898 ^&^	9	15	0.316 *
pT3 or pT4	19	48		24	24	
Lymph nodes metastasis						
pN0	12	43	0.811 ^&^	20	23	0.702 *
pN1 and pN2	12	24		10	14	
Distant metastases						
pM0	20	58	1.000 ^#^	26	32	0.729 ^&^
pM1	4	14		7	7	
Stage						
pTNM I or II	11	43	0.235 *	20	32	0.888 *
pTNM III or IV	13	29		13	16	
Lymphocyte infiltration						
absent	15	39	0.517 *	17	22	0.781 *
present	9	32		15	17	
Venous invasion						
absent	9	29	0.810 *	13	16	0.888 *
present	15	43		20	23	

* χ^2^ test, ^#^ χ^2^ test with Yates’ correction, and ^&^ V^2^ test.

**Table 3:** Relationship between *ABCG2* gene expression and selected clinicopathological factors.

Feature	*ABCG2* Gene Expression Level			
	Min.	Median	Max.	*p*-Value *
Age				
up to 60 y	0.63	0.03	17.64	0.718
over 60 y	0.68	0.02	14.63	
Sex				
female	0.67	0.02	16.38	0.403
male	0.64	0.03	17.64	
Tumour localization				
rectum	0.66	0.04	14.63	0.911
colon	0.67	0.02	17.64	
Histological type				
adenocarcinoma tubulare	0.63	0.02	17.64	0.694
adenocarcinoma mucinosum	0.93	0.12	6.58	
Histological grade				
G1 or G2	0.63	0.04	16.38	0.362
G3	0.83	0.02	17.64	
Depth of tumour invasion				
pT1 or pT2	0.61	0.04	14.63	0.655
pT3 or pT4	0.67	0.02	17.64	
Lymph node metastasis				
pN0	0.65	0.03	17.64	0.773
pN1 and pN2	0.65	0.02	16.38	
Distant metastases				
pM0	0.69	0.03	17.64	0.149
pM1	0.55	0.02	8.58	
Stage				
pTNM I or II	0.67	0.03	17.64	0.799
pTNM III or IV	0.65	0.02	16.38	
Lymphocyte infiltration				
absent	0.78	0.04	16.38	0.228
present	0.58	0.02	14.60	
Venous invasion				
absent	0.67	0.03	17.64	0.798
present	0.65	0.02	11.19	

* Mann–Whitney U-test.

**Table 4:** Overall survival concerning clinicopathological features, ABCG2 protein level and *ABCG2* gene expression.

Feature	Overall Survival	
	Number of Deaths (%)	*p*-Value *
Age		
up to 60 y	22 (48.9)	0.288
over 60 y	30 (60.0)	
Sex		
female	27 (54.0)	0.763
male	25 (55.6)	
Tumour localization		
rectum	24 (68.6)	0.141
colon	28 (47.5)	
Histological type		
adenocarcinoma tubulare	45 (54.9)	0.915
adenocarcinoma mucinosum	7 (53.9)	
Histological grade		
G1 or G2	33 (50.0)	0.112
G3	19 (65.5)	
Depth of tumour invasion		
pT1 or pT2	12 (41.4)	0.041
pT3 or pT4	40 (61.6)	
Lymph node metastasis		
pN0	23 (41.8)	0.001
pN1 and pN2	25 (71.4)	
Distant metastases		
pM0	34 (44.2)	<0.001
pM1	18 (100.0)	
Stage		
pTNM I or II	22 (40.7)	<0.001
pTNM III or IV	30 (73.2)	
Lymphocyte infiltration		
absent	34 (63.0)	0.036
present	17 (42.5)	
Venous invasion		
absent	17 (44.7)	0.070
present	35 (61.4)	
ABCG2 protein expression		
absent	10 (43.5)	0.236
present	42 (58.3)	
low	24 (72.7)	0.077
high	18 (46.2)	
*ABCG2* expression level		
low	36 (63.2)	0.080
high	14 (42.4)	

* Log-rank test.

## Data Availability

Links to publicly archived datasets analysed: https://www.oncomine.org (accessed on 1 January 2022), http://timer.cistrome.org (accessed on 1 January 2022), https://tnmplot.com (accessed on 1 January 2022), http://ualcan.path.uab.edu/index.html (accessed on 29 March 2023), http://dna00.bio.kyutech.ac.jp/PrognoScan/index.html (accessed on 1 January 2022), http://www.cbioportal.org (accessed on 4 February 2023), https://string-db.org/ (accessed on 1 January 2023), http://gepia2.cancer-pku.cn/ (accessed on 29 March 2023), https://www.proteinatlas.org/ (accessed on 29 March 2023), https://www.mexpress.be/ (accessed on 22 May 2023).

## Supplementary Materials

The supporting information can be downloaded at: https://www.mdpi.com/article/10.3390/ijms241310539/s1.
