# Supplementary material for: ABCG2 Gene and ABCG2 Protein Expression in Colorectal Cancer—In Silico and Wet Analysis
Source: Int J Mol Sci. 2023 Jun 23;24(13):10539. doi: 10.3390/ijms241310539 (PMC10341438; doi:10.3390/ijms241310539)
Supplement: Supplementary file 1 [file ijms-24-10539-s001.zip › ijms-2439789-supplementary.pdf]

Supplementary Table S1. Characteristics of the study group.

Feature	Number of Deaths (%)
Age	
up to 60 yrs	45 (46.9)
over 60 yrs	51 (53.1)
Gender	
woman	50 (52.1)
man	46 (47.9)
Tumour localization	
rectum	35 (36.5)
colon	61 (62.5)
Histological type	
adenocarcinoma tubulare	83 (86.5)
adenocarcinoma mucinosum	13 (13.5)
Histological grade	
G1	9 (9.4)
G2	58 (60.4)
G3	29 (30.2)
Depth of tumour invasion	
pT1	4 (4.2)
pT2	25 (26.0)
pT3	56 (58.3)
pT4	11 (11.5)
Lymph nodes metastasis	
pN0	55 (57.3)
pN1	18 (18.8)
pN2	18 (18.8)
NA	5 (5.2)
Distant metastases	
pM0	78 (81.3)
pM1	18 (18.8)
Stage	
pTNM I	27 (28.1)
pTNM II	27 (28.1)
pTNM III	24 (25.0)
pTNM IV	18 (18.8)
Lymphocyte infiltration	
absent	54 (56.3)
present	41 (42.7)
NA	1 (1.0)
Venous invasion	
absent	38 (39.6)
present	58 (60.4)
Adjuvant chemotherapy	
yes	35 (36.5)
no	46 (47.9)
NA	15 (15.6)

NA – data not available.
